# Tellurite and selenite processing by tellurite resistant marine microbes

**DOI:** 10.1128/aem.00881-25

**Published:** 2025-10-01

**Authors:** Patrick Ollivier, Thomas Hanson, Emmanuel Tessier, David Amouroux, Thomas Church

**Affiliations:** 1 BRGM 52810, Orléans, France; 2 School of Marine Science and Policy and Delaware Biotechnology Institute, University of Delaware 5972, Newark, Delaware, USA; 3 Université de Pau et des Pays de l’Adour, E2S UPPA, CNRS, IPREM, Institut des Sciences Analytiques et de Physico-chimie pour l'Environnement et les Materiaux 131730, Pau, France; Georgia Institute of Technology, Atlanta, Georgia, USA

**Keywords:** tellurite, selenite, marine microbes, resistance, nanoparticles, volatile compounds

## Abstract

Understanding microbial transformations of the group VIa/16 metalloids tellurium and selenium is important for the remediation of contaminated environments and has been proposed as a green route for Se/Te nanoparticle synthesis. We previously isolated several strains of aerobic tellurite resistant marine yeast and bacteria. Here, we explored the capability of these strains to metabolize selenite and mixtures of tellurite and selenite to quantify fate and identify volatile metabolic products. The experimental results indicate that selenite is metabolized differently than tellurite by the yeast *Rhodotorula mucilaginosa* and bacteria *Bacillus* spp. and *Virgibacillus halodenitrificans*. The production of volatile Se compounds appears to be positively correlated with selenite resistance. However, selenite fate, e.g., the proportion of volatilized or precipitated Se, was not predictable from tellurite resistance or fate of the same strain. Under non-aerated conditions, when cultures were provided mixtures of selenite and tellurite, tellurite strongly influenced the fate of selenite and the types of volatile products made. Tellurite in oxyanion mixtures appears to strongly inhibit Se volatilization and drive speciation to less complex Se volatiles. Mixtures boosted the production of Te and Se precipitates by *Bacillus* sp. strain 6A and the production of Te precipitates by *Rhodotorula mucilaginosa* strains 13B and decreased the production of both volatile Te and Se compounds. Dimethylselenide and dimethyltelluride are acutely toxic by inhalation and oral exposure, so understanding their production is a key consideration in any biologically based manufacture of Se/Te containing nanoparticles.

**IMPORTANCE:**

Many microbes are remarkably resistant to high concentrations of both selenite and tellurite while producing less toxic and bioavailable elemental forms, providing opportunities for the remediation of contaminated environments and green biosynthesis of Se/Te nanoparticles. The toxicity of volatile tellurite and selenite compounds produced during microbial processing may limit the development of remediation and biosynthesis technologies. The precise biochemical mechanisms governing Te and Se fate are still unclear. The data presented here demonstrate that combining Se and Te influenced the tolerance of marine microbes (*Rhodotorula mucilaginosa* 13B and *Bacillus* sp. strain 6A) to tellurite, significantly increasing precipitation as a product while limiting volatilization with the implication that combined Se/Te microbial remediation and/or nanoparticle synthesis may be less problematic than single element processes.

## INTRODUCTION

The group 16/VIA elements Oxygen and Sulfur are well known for their utilization by microbes in reduction/oxidation reactions spanning oxidation states of 0 to −2 for Oxygen and +6 to −2 for Sulfur. The heavier group 16 elements Selenium and Tellurium display a similar range of oxidation states and chemical species relative to Sulfur: +6 (selenate/tellurate), +4 (selenite/tellurite), 0 (elemental selenium and tellurium), and −2 (selenide/telluride). Of these species, selenite and tellurite are highly toxic. However, many microbes have shown a remarkable ability to resist high concentrations of both selenite and tellurite while reducing them to less toxic and bioavailable chemical forms (1–8). Reduction to the elemental state and methylation of reduced species are thought to be important for resistance, but the precise biochemical mechanisms involved are still matter of debate and there is considerable complexity and variation between different microbes (9).

Both Se and Te have industrial applications, for example, in photovoltaic technologies and rechargeable batteries. Microbial reduction of Se and Te has attracted great interest for diverse applications as an alternative to physico-chemical methods for the production of useful materials or remediation of waste streams. For example, Se and Te reducers have been applied in wastewater treatment to remediate Se and Te contaminated environments (10–20). The biosynthesis of Se and Te nanoparticles (NPs) has also received extensive attention (21–31). Se and Te NPs have unique physical and chemical properties including photoelectric and semiconducting characteristics, with biological antimicrobial, antioxidant, and anticancer activity (14, 32–36). Microbial NP synthesis methods are attractive because they are cost-effective, environment-friendly compared to available methods and generate nanomaterials with properties unattainable by conventional physical/chemical methods, e.g., increased stability and decreased toxicity (14, 37–41).

Microorganisms reduce both selenite and tellurite separately to elemental Se and elemental Te and simultaneous biological reduction of both Se and Te oxyanions has been reported (23, 31, 42–46). Mixtures of both oxyanions may influence microbial reduction of the other oxyanion and *vice-versa*. For instance, selenite increased the aerobic tellurite reduction rate of *Duganella violacienigra* by 13-fold (43). Furthermore, microbes produced mixed Se–Te nanoparticles when grown with selenite/tellurite (43–45). Compared to pure Te- or Se-NPs, Se-Te composites offer unique semi-conductive and optical properties, as well as enhanced electrical and magnetic resistance properties with potential applications in advanced electronic and optoelectronic devices (47, 48).

Previously, we showed that the marine yeast *Rhodotorula mucilaginosa* (strains *Rm*-1A, *Rm*-13B, and *Rm*-30B) and Gram-positive marine bacteria of the family Bacillaceae (*Bacillus* spp. strains *Bac_*sp-6A and *Bac*_sp-28A, and *Virgibacillus halodenitrificans* strain 14B, *Vh*-14B) produce large amounts of intracellular Te NPs at moderate temperature in aerated and non-aerated liquid cultures (49, 50). For example, *R. mucilaginosa* strains converted ~95% of 0.7 mM tellurite to particulate Te NPs. *R. mucilaginosa Rm*-13B was also capable of reducing a substantial amount of selenite to form amorphous elemental Se NPs (51). These isolates precipitate greater quantities of Te than gram-negative bacteria making them potentially attractive systems for the synthesis of Se, Te, or mixed NPs.

However, these marine yeast and bacteria volatilized methylated Te and S species simultaneously with precipitation (49, 50). Various microorganisms are capable of volatilizing inorganic Se and Te compounds (52), but these byproducts are not considered in literature focused on microbial NP production. While alkylated Se and Te are much less toxic than selenite or tellurite, the dimethyl species are still classified as acutely toxic chemicals. The toxicity of other volatile alkylated forms is not yet established. An important consideration for “green” chemical and microbial technologies is preventing or limiting toxic byproducts of processes.

Here, we documented Se and Te fate when the marine yeast and bacteria strains above are grown with only selenite or selenite/tellurite mixtures. Different Se to Te ratios (10:1, 1:1, or 1:10 ratios) were tested to with particular emphasis on the modulation of volatile species production from mixed oxyanion cultures to determine if these conditions boost the Te and Se NP production and generate less volatile byproducts.

## RESULTS

### Resistance to selenite

**Fig 1:**
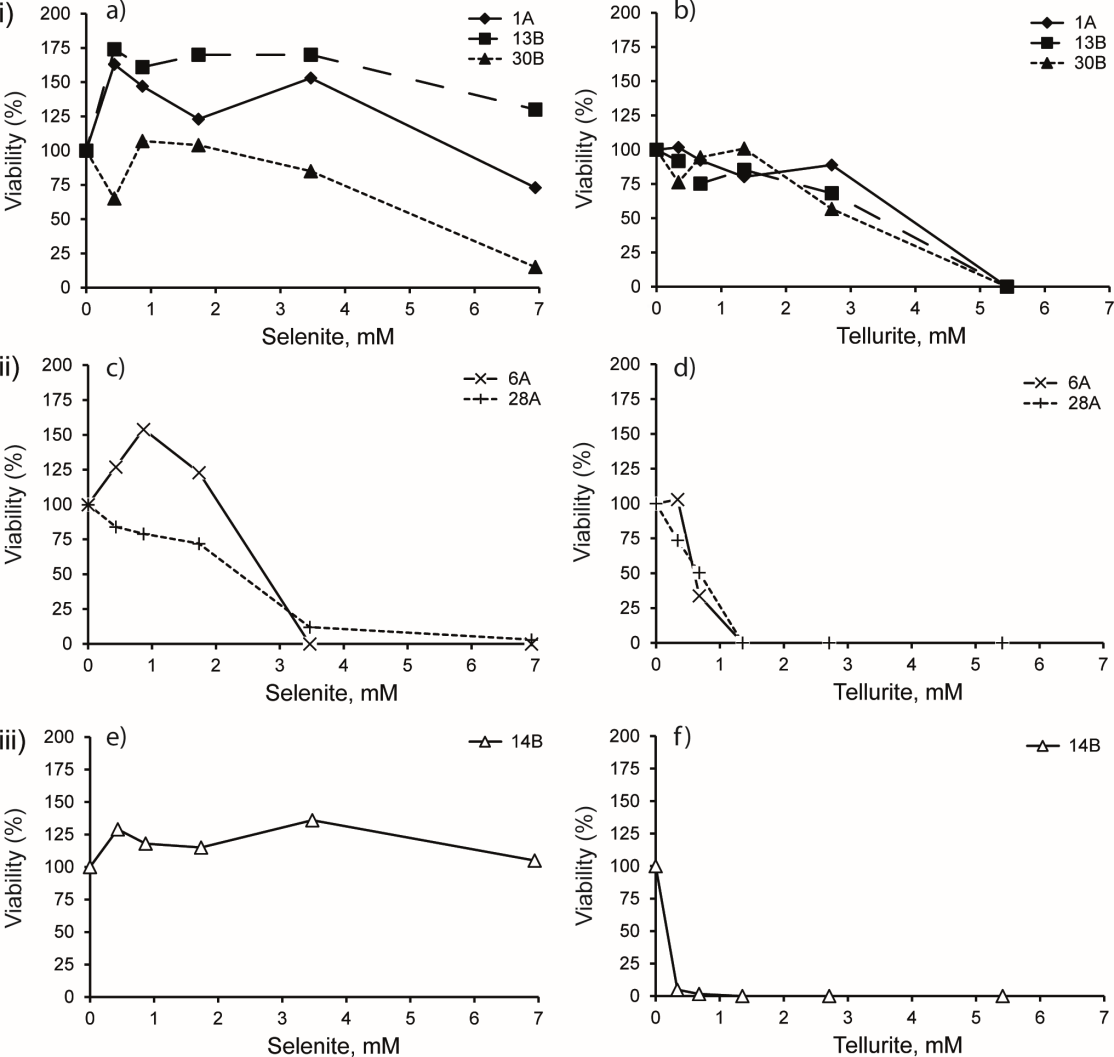
Resistance of strains to various concentrations of sodium selenite (**a**, **c**, and **e**; our data) and sodium tellurite (**b**, **d**, and **f**; data from reference 49) on LB-marine plates. The viable population observed on LB-plates without tellurite or selenite was defined as 100% viability. (i) Cluster 1 strains *Rm*-1A, *Rm*-13B, and *Rm*-30B. (ii) Cluster 2 strains *Bac_*sp-6A and *Bac*_sp-28A. (iii) Cluster 3 strain *Vh*-14B. Symbols for each strain are noted in the figure. Data points are the average of the results for two independent experiments for each strain.

The strains studied here (Table 1) were originally clustered based on their tellurite resistance (Fig. 1b, d and f; data from reference 49 for ease of comparison). Highly tellurite resistant strains *R. mucilaginosa* (*Rm*-1A, *Rm*-13B, *Rm*-30B) and moderately tellurite resistant *Bacillus* spp. strains (*Bac*_sp-6A, *Bac*_sp-28A) are highly and moderately selenite resistant (Fig. 1a and c). They are quantitatively more resistant to selenite and the relative resistance clustering is the same (Table 1). However, *Virgibacillus halodentirificans* strain *Vh*-14B displayed the highest level of selenite resistance (*Vh*-14B, Fig. 1e), up to 7 mM. This starkly contrasts with its weak tellurite resistance (Fig. 1f). Most strains displayed greater viability at low selenite concentrations relative to no added selenite reflecting selenium’s documented role as a micronutrient (8).

**TABLE 1:** Strains and identifiers used in this work^*a*^

Organism	Abbreviations	Tellurite-resistance cluster
*Rhodotorula mucilaginosa*	*Rm*-1A, -13B, -30B	I-High (>2 mM)
*Bacillus* spp.	*Ba_*sp-6A, -28A	II-Moderate (1–2 mM)
*Virgibacillus halodenitrificans*	*Vh*-14B	III-Low (<1 mM)

^*a*^ All strains were isolated from Delaware salt marsh sediments.

### Fate of Se and Te in single oxyanion experiments

**Fig 2:**
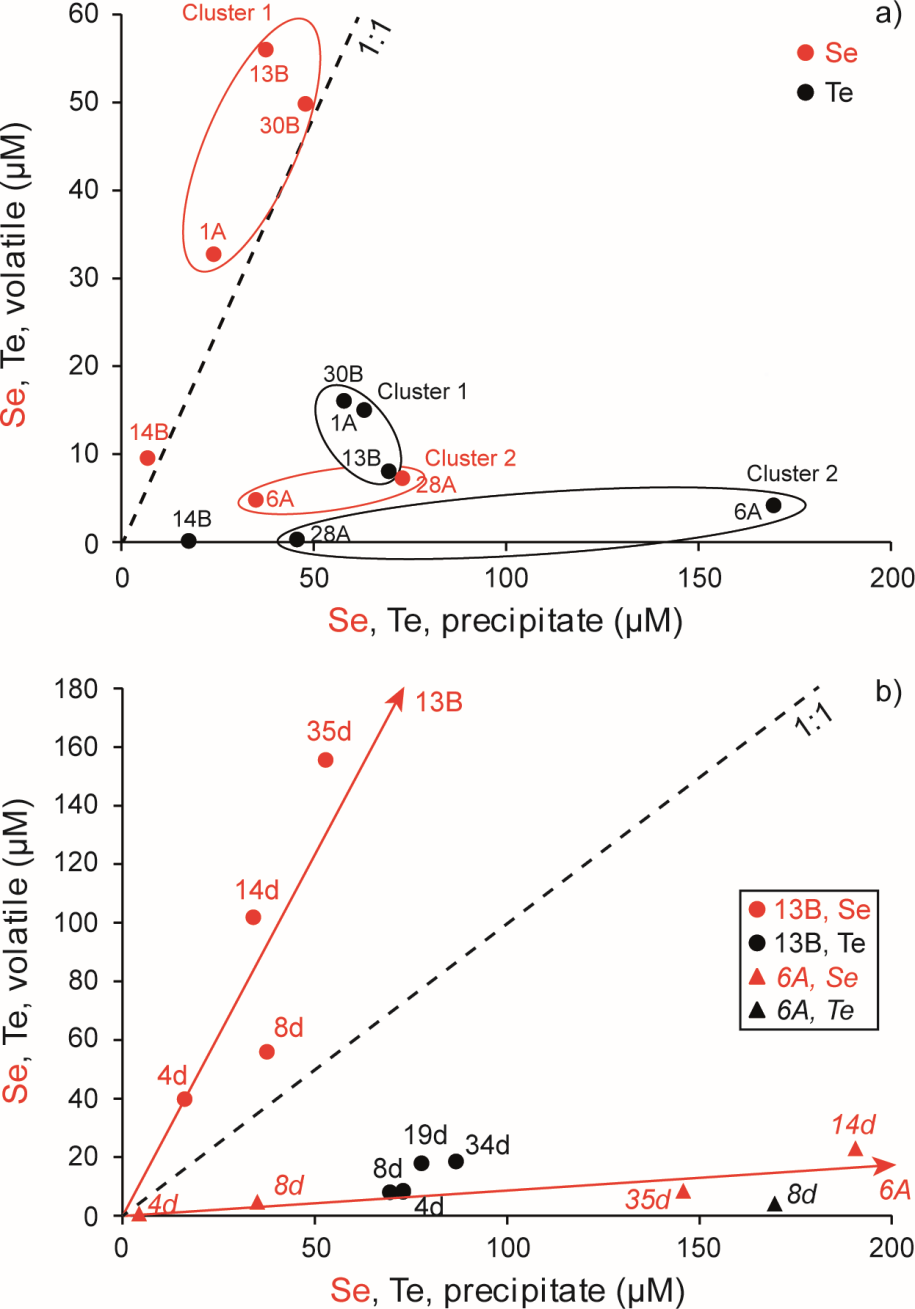
Particulate and volatile Se and Te in cultures (10 mL) of cluster 1 strains (*Rm*-1A, *Rm*-13B, and *Rm*-30B), cluster 2 strains (*Bac*_sp-6A and *Bac*_sp-28A), and cluster 3 strain (*Vh*-14B) after 8 days of growth with continuous aeration (**a**) and in cultures (10 mL) of strains *Rm*-13B and *Bac_*sp-6A after 4, 8, 14 (or 19) and 34 (or 35) days of growth, with continuous aeration (**b**). A total of 0.68 mM tellurite or 0.68 mM selenite was added to cultures. The data presented were obtained from individual experiments performed for each condition and each strain.

In aerated cultures, Se and Te exhibited distinct fates across strains when provided singly. Cultures grown in medium with selenite formed an orange-red precipitate indicating formation of Se(0) NPs (as previously reported by reference 51). Similarly, black precipitates of Te(0) NPs were observed in the presence of tellurite (consistent with previous observations by references 49 and 50). Highly selenite resistant strains *Rm*-1A, -13B, and -30B volatilized more Se than they precipitated (Fig. 2a, red symbols) with all points falling above the 1:1 line for volatile:precipitate. *Rm-*13B was the most active, when supplied with 676 µM selenite it converted 5.9% (38 µM) to solids and 8.8% (56 µM) to volatiles after 8 days of growth. Moderately selenite resistant strains *Bac*_sp-6A and -28A produced more precipitates than volatiles. *Bac*_sp-28A supplied with the same selenite concentration converted 11.0% (73 µM) to solids vs 1.1% (7 µM) to volatiles in the same time. Te precipitation was strongly favored over volatilization (Fig. 2a, black symbols) as all data points are far below the 1:1 volatile:precipitate line. *R. mucilaginosa* strains produced the highest amounts of Te volatiles: 1.2%–2.4% (8–16 μM) of the tellurite supplied. Strain *Bac_*sp-6A converted the most tellurite to solids: 25% (170 μM) of the starting amount. *Vh-*14B behaved much differently than other strains. This weakly tellurite-resistant, but highly selenite-resistant, strain converted the least selenite and tellurite to solids and volatilized much less Se than less selenite resistant strains. Even so, Se volatilization was slightly favored over precipitation at 8 days of growth.

Time dependence of Se and Te fate was followed in the highest Se-volatilizing strain, *Rm*-13B, and the highest Te-precipitating strain, *Bac*_sp-6A. *Rm*-13B sustained high rates of Se volatilization relative to precipitation over time (Fig. 2b; Fig. SI-1, red circles; 23% [156 µM] of total Se [676 µM] was converted to volatile forms after 35 days of growth) indicated by the data points diverging further from the 1:1 line with increasing time. Se precipitation was essentially complete after 8 days. *Rm*-13B precipitated Te more rapidly, most activity occurred in 4 days, and extensively than Se (Fig. 2b; Fig. SI-1, black circles); the amount of Se precipitated after 35 days was less than Te precipitated at 4 days. Volatilization of Te was much less extensive than Se, but *Rm*-13B did appear to continuously volatilize Te over time indicated by greater separation from the precipitate axis (Fig. 2b, black circles). Strain *Bac_*sp-6A displayed a complicated temporal pattern of Se metabolism (Fig. 2b red triangles). Se precipitation was strongly favored over volatilization for the first 14 days, but at 35 days, both the amount of solid and volatile Se had decreased relative to day 14. These experiments use sacrificial cultures rather than time series sampling of the same culture. Strain *Bac_*sp-6A precipitated Te about fourfold faster than Se, ~18 µM Te day^−1^ vs 4.4 µM Se day^−1^.

**Fig 3:**
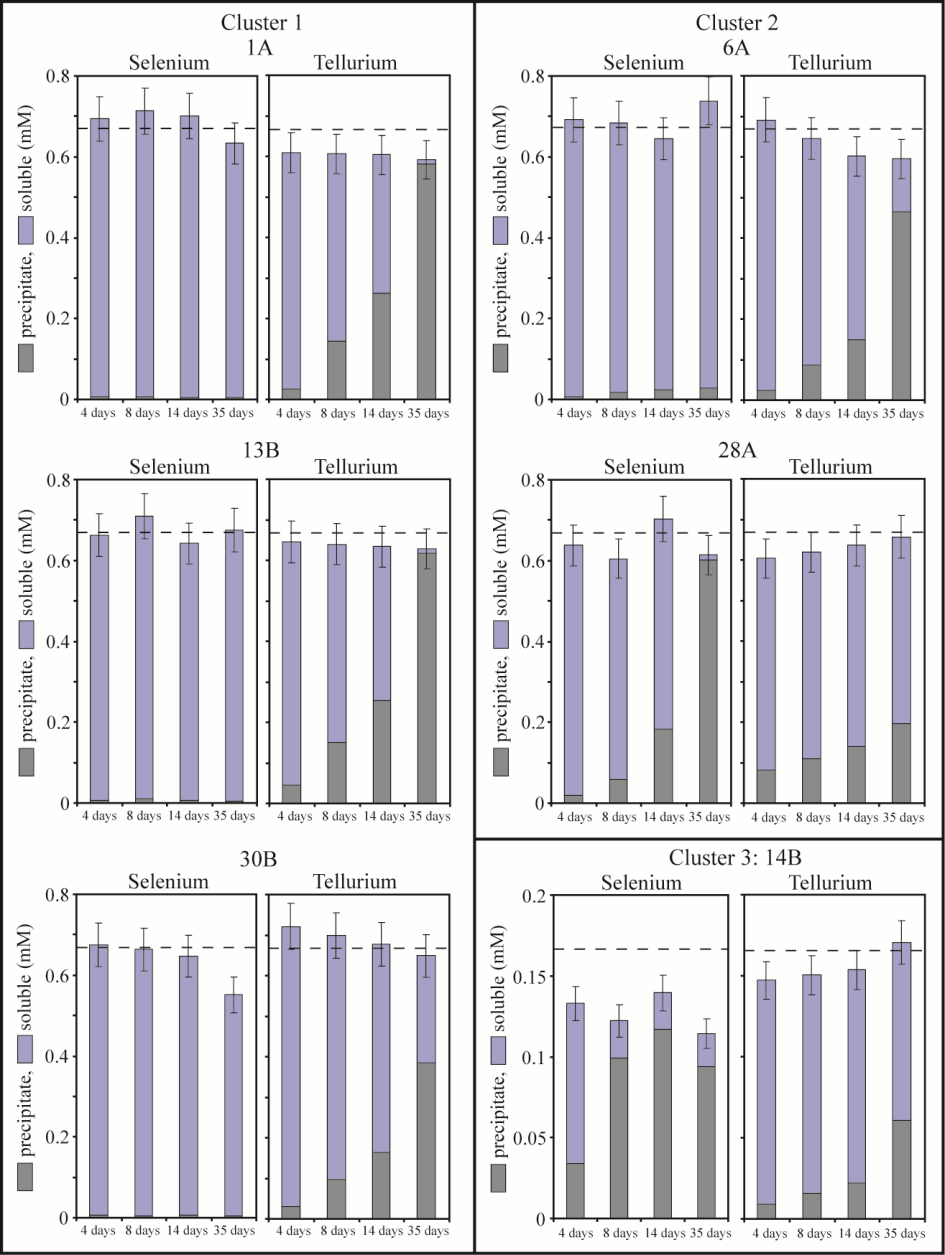
Soluble (light bars) and particulate (dark bars) Te and Se in strains in non-aerated liquid cultures exposed to SeO_3_^2−^ or TeO_3_^2−^ independently. A total of 0.68 mM SeO_3_^2−^ or 0.68 mM TeO_3_^2−^ was added to cultures of cluster 1 strains and cluster 2 strains, while the culture of strain *Vh*-14B received 0.17 mM SeO_3_^2−^ or 0.17 mM TeO_3_^2−^ (cluster 3), denoted by the dashed lines in each panel. The data presented correspond to the average of the results of two independent experiments conducted for each strain. Bars are the mean of four measurements (± the standard deviation). Te data originally appeared in reference 49.

Non-aerated cultures processed Se very differently than aerated cultures. Very little Se was volatilized as the total (solid + soluble) Se recovered overlaps within error of the total selenite supplied (Fig. 3). Highly resistant strains *Rm*-1A, *Rm*-13B, and *Rm*-30B appeared to not react with selenite as <1% of added Se was recovered as solids. Highly resistant *Vh*-14B is the only one that displays evidence of volatilization as incomplete mass balance of Se; the sum of soluble and solid is less than the selenite provided. Strain *Vh*-14B and the moderately selenite resistant *Bac*_sp-28A precipitated significant Se continuously under non-aerated conditions (Fig. 3). Similarly, all strains precipitated Te under non-aerated conditions, with strains *Bac*_sp-28A and *Vh*-14B precipitating more Se than Te. These behaviors must be well understood to effectively use these strains for NP production.

### Volatile Te and Se species in single oxyanion experiments

**Fig 4:**
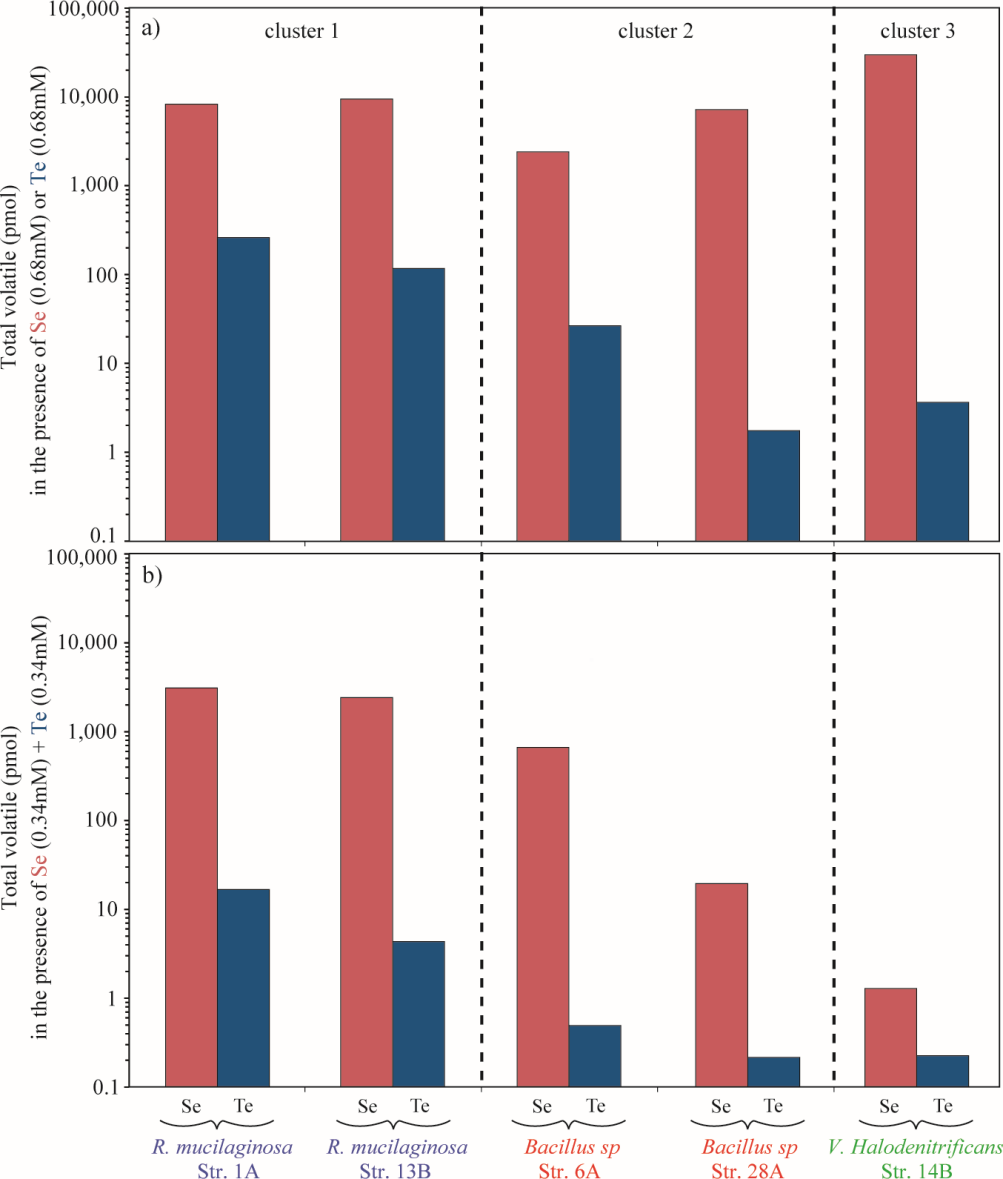
Total Se and Te volatil production in cultures amended with (**a**) Se (0.68 mM) or Te (0.68 mM) independently and (**b**) with a mixture Se (0.34 mM) + Te (0.34 mM). The data presented were obtained from individual experiments performed for each condition and each strain.

Volatile species were not detected in non-aerated culture headspace samples by standard GC-MS. Therefore, GC-inductively coupled plasma (ICP)-MS analysis was used in non-aerated culture for its higher sensitivity. GC-ICP-MS detected multiple forms of volatile Se and Te species in single oxyanion experiments. Se volatilization is more prevalent than Te volatilization (Fig. 4a), with total volatile Se compounds produced ranging from 33-fold higher (*Rm*-1A) to >10,000-fold higher (strain *Vh*-14B) than volatile Te compounds. The total amount of volatile Se detected in the culture media of strains belonging to clusters 1 and 2 accounts for less than 1% (ranging from 0.1% to 0.7%) of the total Se supplied, explaining why volatiles were not detected in bulk experiments (Fig. 3). *Vh*-14B exhibited the highest production of volatile Se (~31 nmol Se), representing 2.3% of the total Se supplied. Tellurite resistance is linked with tellurite volatilization. Highly tellurite resistant strains *Rm*-1A and *Rm*-13B volatilized the most Te, followed by moderately resistant strains *Bac*_sp-6A and *Bac*_sp-28A, with weakly tellurite resistant strain *Vh*-14B volatilizing the least Te (Fig. 4a). Se volatilization was less variable across strains, but the most highly selenite resistant strain *Vh*-14B volatilized the most Se followed by strains *Rm*-1A and *Rm*-13B, with the least selenite-resistant strains *Bac*_sp-6A and *Bac*_sp-28A volatilizing the least Se.

DMTe was the most-abundant volatile tellurium species detected and was produced by all strains in non-aerated liquid cultures (Fig. SI-2a). DMDTe, when present, was less abundant than DMTe; it was not produced by moderately resistant strains. Other Te compounds are detected in low quantity such as DMTTe (CH_3_TeTeTeCH_3_), MTeH? (CH_3_TeH; identification has to be confirmed), and DEtTe (C_4_H_10_Te) (data not shown). The mixed species dimethyltellurenyl sulﬁde (DMTeS; CH_3_TeSCH_3_) is found in cultures of all strains except for strain *Bac*_sp-28A. These results are consistent with prior work demonstrating volatile alkylated tellurium and sulfur compound production by these cultures (49).

Volatile Se species were more diverse than volatile Te species in non-aerated cultures. Highly resistant strains *Rm*-1A and *Rm*-13B produce predominantly DMDSe (CH_3_SeSeCH_3_), while *Vh*-14B volatiles were dominated by DMSeS (CH_3_SeSCH_3_). Moderately resistant strains *Bac*_sp-6A and *Bac*_sp-28A produce the mixed species DMDSSe (CH_3_SeSSCH_3_) and DMSeS that account for 87% to 95% of the total amount of volatile Se produced (Fig. SI-2b). DMTSe and DMDSeS (CH_3_SeSeSCH_3_) are only detected at low levels. All of these volatile Se species have previously been identified in the environment or in headspace above cultures of strains in laboratory experiments (53–55). Compared to continental aquatic ecosystems where DMSe was the dominant volatile compound, marine environments and sulfate-enriched waters produce mixed sulfur-Se compounds, DMDSe or DMSeS as major volatiles (55–60). These results are consistent with the marine origins of the strains studied here.

### Fate of Se and Te in mixed oxyanion experiments

Se and Te fate was determined in mixed oxyanion cultures. Different ratios of selenite to tellurite were provided to examine how the presence of each oxyanion affects the fate of the other.

In aerated cultures, tellurite fate was unaffected by the presence of selenite in strains *Rm*-13B and *Bac_*sp-6A (Fig. SI-3). In contrast, Se volatilization and Se-precipitation were markedly reduced in both strains when tellurite was present at >1:1 molar ratio. Thus, tellurite appears to have a greater effect than selenite in aerated cultures.

**Fig 5:**
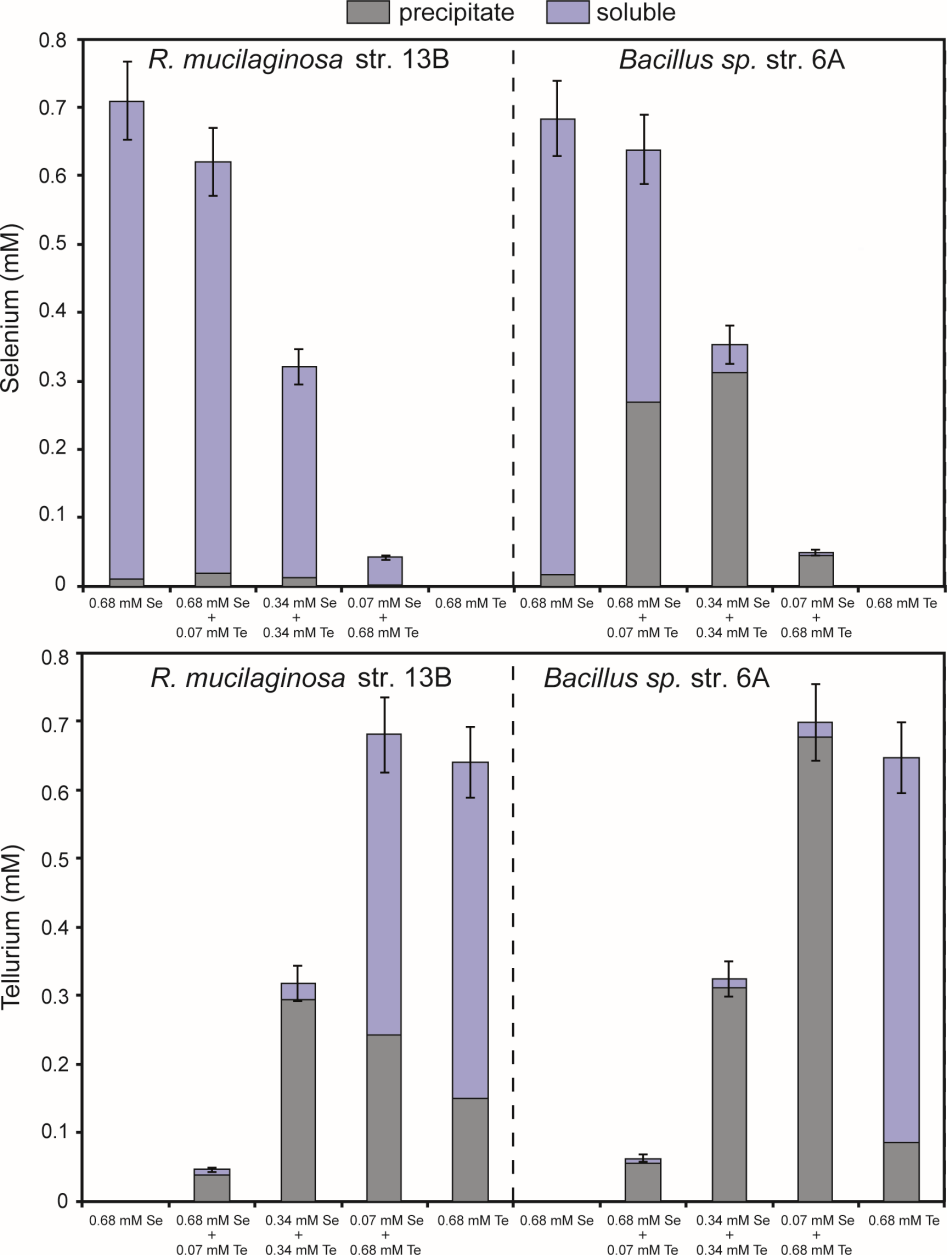
Soluble (blue bars) and particulate (dark bars) Se (top) and Te (bottom) in strains *Rm*-13B and *Bac_*sp-6A, after 8 days of growth, in non-aerated liquid cultures exposed to SeO_3_^2−^ (0.68 mM), TeO_3_^2−^ (0.68 mM), and various mixtures SeO_3_^2-^ and TeO_3_^2-^ [0.68 mM(Te):0.068 mM(Se), 0.34 mM(Te):0.34 mM(Se) or 0.068 mM(Te):0.68 mM(Se) ratios, i.e., 10:1, 1:1, or 1:10 ratios]. The data presented were obtained from individual experiments performed for each condition and each strain.

As with single oxyanion non-aerated experiments, there was no evidence of bulk volatilization of Se or Te in non-aerated mixed oxyanion experiments. For strain *Rm*-13B, high tellurite:selenite inhibited Se precipitation (Fig. 5). But selenite increased Te precipitation: Te solids were twofold higher at 1:1 molar ratio compared to 0.68 mM tellurite alone. For strain *Bac*_sp-6A, Se or Te precipitation was strongly enhanced by the other anion even at low molar ratios. After 8 days of growth, 42% of the Se was precipitated in 10:1 selenite:tellurite cultures compared to <3% in cultures containing selenite only. Similarly, 97% of added Te precipitated in 10:1 tellurite:selenite cultures containing compared ~13% in cultures containing tellurite alone. Cultures with 1:1 anion ratios precipitated 88% of Se and 96% of Te.

Conversely, for strains *Bac*_sp-28A and *Vh*-14B, in non-aerated cultures, tellurite addition decreased Se precipitation and *vice versa* (except for 14B; Fig. SI-4). This is significant because these strains were thought to be strong candidates for NP production, given that they produce the highest levels of precipitated selenium in the presence of selenite.

### Volatile Te and Se species in mixed oxyanion experiments in non-aerated cultures

**Fig 6:**
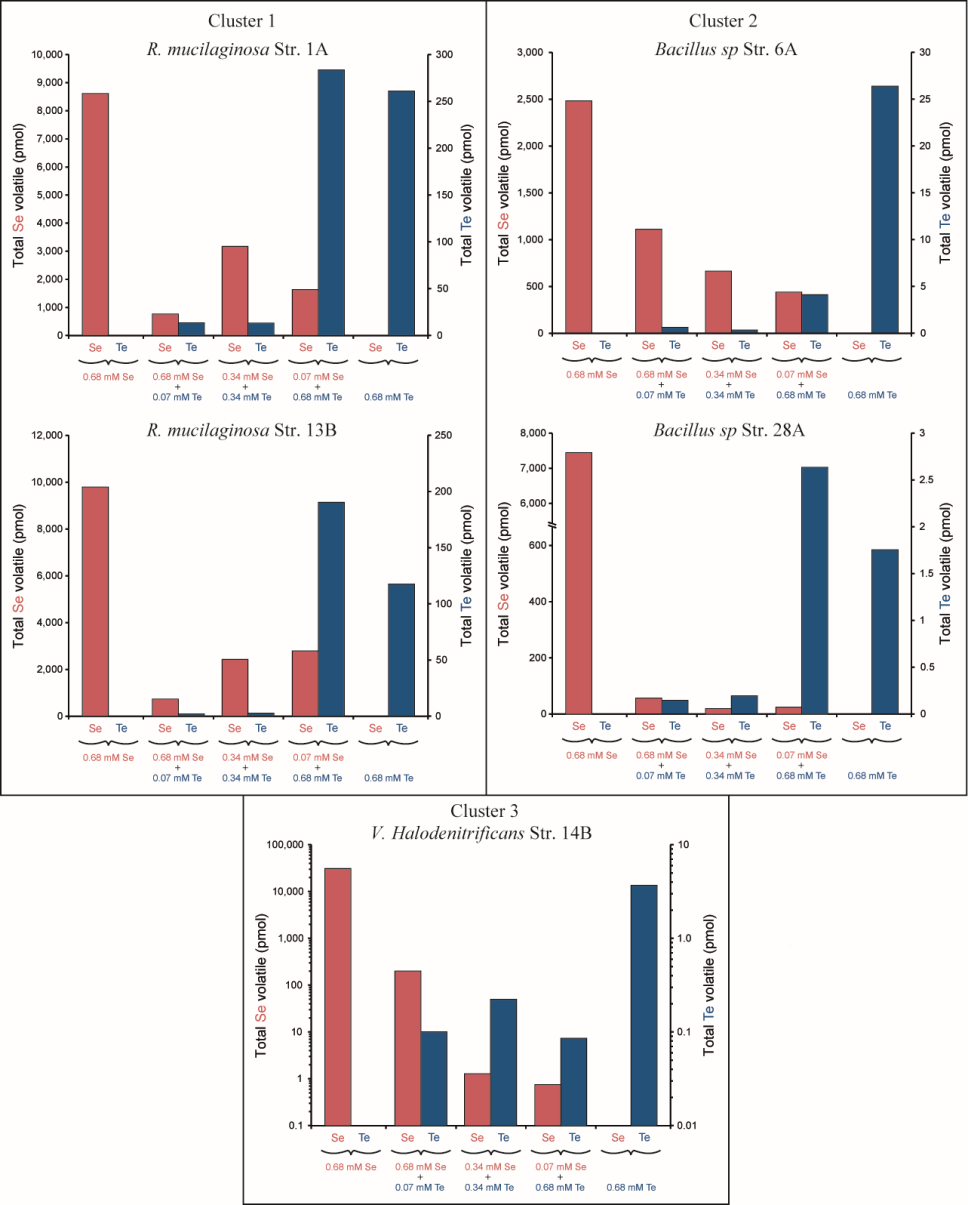
Total Se (red) and Te (blue) volatile production in headspace of non-aerated liquid cultures amended with 0.68 mM SeO_3_^2-^, 0.68 mM TeO_3_^2-^, and various mixtures SeO_3_^2-^ and TeO_3_^2-^ [0.68 mM(Te):0.068 mM(Se), 0.34 mM(Te):0.34 mM(Se), or 0.068 mM(Te):0.68 mM(Se) ratios, i.e., 10:1, 1:1, or 1:10 ratios]. The data presented were obtained from individual experiments performed for each condition and each strain.

Non-aerated cultures with tellurite and selenite mixtures produced much lower levels of volatile compounds across all strains while volatilizing more Se than Te. The reduction in volatilization was observed with low-level addition. For example, the 10:1 selenite:tellurite mixture reduced Se volatilization relative to selenite-only cultures by ~150-fold for strain 14B, ~130-fold for strain *Bac*_sp-28A, ~10-fold for *R. mucilaginosa* strains (1A and 13B), and ~2-fold for strain *Bac_*sp-6A (Fig. 6). Selenite appeared less inhibitory. The 10:1 tellurite:selenite mixture reduced Te volatilization by ~40-fold for strain *Vh*-14B and ~6-fold for strain *Bac_*sp-6A, while 1:1 ratios inhibited Te volatilization by *Rm*-1A (20-fold), *Rm*-13B (42-fold), and *Rm*-30B (4-fold) compared to that observed in the absence of Se.

The presence of tellurite modified volatile Se compound speciation in a complicated pattern (Fig. SI-5, left panels). In strains *Rm*-1A and *Rm*-13B, increasing tellurite:selenite ratios progressively favored DMSe production instead of DMDSe and DMSSe, the dominant species in the absence of tellurite. For example, strain *Rm*-13B produced 3% DMSe in the absence of tellurite, 40% at 1:10 tellurite:selenite which increased to 99% at 10:1 tellurite:selenite. Over the same range, DMDSe decreased from 45% of volatile Se at 1:10 tellurite:selenite to <0.5% at 10:1 tellurite:selenite (Fig. SI-6). *Bac*_sp-6A also increased DMSe and limited production of the mixed species DMSSe and DMDSSe with increasing tellurite (Fig. SI-5). DMSSe and DMDSSe were 94% of Se volatiles produced by *Bac*_sp-6A in the absence of tellurite (65% DMDSSe + 29% DMSSe), while DMSe was <1%. At 1:1 tellurite:selenite, DMSe was 60% of the total Se volatiles produced by *Bac*_sp-6A with mixed species constituting <20% (Fig. SI-6). Trends for other strains are shown in SI-1.

For Te, responses to selenite were more subtle. Total amounts of Te volatiles decreased as the tellurite:selenite ratio decreased (Fig. SI-5, right panels). For *Rm-*1A, *Rm*-13B, and *Bac*_sp-6A, decreasing tellurite:selenite shifted Te volatiles from DMTe to DMDTe or others (e.g., DMTTe, MTeH), but this was reversed at the lowest tellurite:selenite ratio (Fig. SI-7). Mixed volatiles containing both Te and S were consistently low in *Rm-*1A, *Rm*-13B, and *Bac*_sp-6A. In contrast, volatiles containing both Te and S increased in relative abundance with decreased tellurite:selenite ratios in *Bac*_sp-28A and *Vh-*14B. Finally, a mixed species with Se and Te (DMSeTe) is found at very low levels (<1%) in the headspace of cultures of all strains except for *Vh-*14B.

As a general rule, tellurite in oxyanion mixtures displayed a stronger effect on Se volatilization and speciation than the reverse. Tellurite appears to strongly inhibit Se volatilization and drive speciation to lighter, less complex Se volatiles, e.g., DMSe.

## DISCUSSION

We have demonstrated that aerobic tellurite resistant marine microbes also precipitate and volatilize Se under the same conditions that they precipitate and volatilize Te. Our previous data on tellurite resistance were a reasonable predictor of selenite resistance except for *V. denitrificans* strain 14B, which was remarkably selenite resistant while only weakly tellurite resistant. The level of tellurite/selenite resistance was positively correlated with volatilization, e.g., more resistant strains volatilized more Se or Te. However, Te fate in terms of the extent or identity of volatilized species was not predictive of Se fate, particularly when cultures were exposed to mixtures of selenite and tellurite. In mixtures, tellurite strongly inhibited Se volatilization and changed the distribution of volatile Se species. Reducing the amount of such volatile compounds produced during NP synthesis is important, as they have serious health and environmental effects from chronic and acute exposure. This is a key aspect for any “green” process development.

### Differences in the response of strains to selenite and tellurite

All strains are more resistant to selenite than tellurite, consistent with the higher toxicity of tellurite relative to selenite (3). Under aerated conditions, Se was volatilized to a greater extent than Te (Fig. 2). *R. mucilaginosa* strains and strain *Vh*-14B volatilized more Se than was precipitated indicating that volatilization is the main pathway of reduction of selenite. But, non-aerated conditions dramatically reduced Se volatilization and markedly increased Se precipitation by strains *Bac*_sp-28A and *Vh*-14B (Fig. 3), exceeding their Te precipitation under these conditions. This is distinctly different than how these strains process tellurite, where precipitation is always favored over volatilization regardless culture aeration.

Differences in the response of strains to selenite and tellurite are more pronounced in non-aerated liquid cultures (Fig. 3). Cluster 1 strains produce low Se precipitates but high Te precipitates. Even closely related organisms exhibit dramatic differences in Se fate. For example, the two bacterial strains *Bac_*sp-6A and *Bac*_sp-28A differ more than 10-fold in their ability to precipitate Se. Differences in the production of volatile and precipitated compounds over time are also observed. Aerated cultures of *Rm*-13B and *Bac-*sp-6A progressively volatilized Se volatiles over time, while Te volatiles and precipitates were more stable over time (Fig. 2b). Conversely, non-aerated cultures of *Rm*-13B and *Bac-*sp-6A precipitated very little Se while Te precipitation increased with time. These results suggest that accumulation of Te and Se in yeast like *Rhodotorula* and gram-positive bacteria *Bacillus* sp. is probably realized *via* distinct pathways. This is consistent with previous works. For instance, it is shown that *Ochrobactrum* sp. MPV1 most likely exploit two different mechanisms to bioprocess selenite and tellurite (61). Kim et al. (62) show that Te(0) reduction by *Shewanella oneidensis* MR-1 is quite different from selenite reduction. Klonowska et al. (42) show differences both in the localizations of Se(0) and Te(0) deposits and in the effects of various inhibitors (2-*n*-heptyl-4-hydroxyquinolineN-oxide [HQNO], antimycin A, pCMB, and potassium cyanide) on selenite and tellurite reduction processes in *S. oneidensis* suggesting that the reduction mechanisms of selenite and tellurite are distinct processes.

We have previously shown that the extent of tellurite precipitation and volatilization was positively correlated with tellurite resistance (49). Cluster 1 strains (high tellurite resistant) produce high Te precipitates when strain *Vh*-14B (low tellurite resistant) produces low Te precipitates. Regarding Se, strain *Vh*-14B exhibits the highest selenite resistant compared to other strains (Fig. 1). In non-aerated liquid cultures, it has high Se precipitation activity suggesting a link between selenite resistance and Se precipitation. Based on this result, non-aerated growth of *Vh*-14B could represent a promising approach for Se NP synthesis. In aerated liquid cultures, the production of Se precipitates by strain *Vh*-14B is low but previous studies show a strong inhibition of selenite precipitation by exposure of microbes to aerobic conditions (63). This is not valid for strains *Rm*-13B and *Bac*_sp-6A. Continuous aeration promotes Se precipitation in cultures, with an increase with time. In addition, they are highly resistant to selenite, but they produce very low Se precipitates in non-aerated liquid cultures. Furthermore, they are less resistant to tellurite than selenite, but they produce high Te precipitates. Therefore, there is a difference between microbial selenite resistance and Se precipitation. This is consistent with work of Wang et al. (64) who have shown that selenite-reducing efficiency is inconsistent with selenite resistance in bacteria.

On the other hand, resistance seems to be related to volatilization. The production of volatile Se compounds appears to be positively correlated with selenite resistance. Strain *Vh*-14B displays the highest tolerance for selenite and produces the greatest amount and diversity of volatile Se compounds relative to other isolates studied. And it produces limited Te volatiles related to its weak tellurite resistance (49, 50). The production of volatile compounds represents a limitation that hinders the relevance of using strain 14B for NP production.

### Oxyanion mixtures enhance NP production and decrease volatilization

The use of microorganisms to remove Te and Se from water and generate nanomaterials is currently the subject of intensive research (21, 22, 30). Previously, we showed that marine microbes (yeast *Rhodotorula mucilaginosa* and *bacillus* sp.) could precipitate high quantities of intracellular Te-containing nanostructures. Here, we demonstrate that they can precipitate Se. For strains *Rm*-13B and *Bac-*sp-6A, Se precipitation increases with time under continuous aeration, which is of interest for the use of these microbes as a “green” route for the production of Se(0)-containing precipitates (presumably as NPs based on prior work with these strains; 49) and for the remediation of Se-oxyanion. Moreover, we show that the presence of Se in non-aerated liquid cultures containing Te dramatically boosts both the removal of tellurite from solutions and the formation of Te precipitates in strains *Rm*-13B and *Bac_*sp-6A (Fig. 5). Bajaj and Winter (43) have previously reported that the presence of selenite triggers bioreduction of tellurite in liquid cultures of heterotrophic non-halophilic aerobic bacteria. Reduction of tellurite was greatly influenced by the presence of excess selenite. Optimum molar ratio of selenite:tellurite for faster tellurite reduction was 6.5:1 (i.e., 0.51 mM Se:0.08 mM Te). When the concentration of selenite was low (0.063 mM), there were no significant changes in tellurite reduction. Kabiri et al. (65) observed also that the tellurite removal increased significantly when both selenite (100 mM) and tellurite (0.5 mM) (i.e., selenite:tellurite ratio 200:1) were added to the culture media of the halophilic bacterium *Halomonas* sp. strain MAM. Here, we show that an excess of selenite is not necessary to significantly influence the reduction of tellurite in strains *Rm*-13B and *Bac*_sp-6A. Furthermore, in strain *Bac*_sp-6A, the presence of low concentration of tellurite in non-aerated solutions influences the precipitation of Se (Fig. 5). Kabiri et al. (65) did not observed significant changes in Se removal in the presence of Te. Bajaj and Winter (43) also showed selenite reduction in solution was not quantitatively affected at a 16:1 selenite:tellurite ratio. However, they observed the formation of Te-Se NP and concluded that tellurite reduction was stimulated by parallel selenite reduction. Probably, in these studies, the low tellurite and high selenite concentrations in cultures did not make it possible to observe a difference in the concentration of Se in solution following Te-Se precipitation. Here, strain *Bac-*sp-6A shows that Te and Se reduction may not be limited to the formation of Se-Te (1:1) composites as high amounts of Te precipitate in the presence of low Se concentration in liquid cultures and *vice-versa*. Previous studies suggest that selenite increases tolerance of microbes to tellurite, thereby facilitating its reduction (12). Here, we show that Strain *Bac-*sp-6A is of clear interest for Te NP synthesis, as its production does not require a large input of selenite (which increases production costs), and the resulting products would mainly consist of pure Te NPs—with little or no Te-Se composite NPs, unlike in other studies where excess selenium is used to boost Te NP production. This still needs to be further investigated through more detailed SEM or TEM observations. For strain *Rm*-13B, Se precipitation is not influenced by the presence of tellurite showing that mixture favors Te precipitation but not Se or Se-Te precipitation (Fig. 5).

Above, in single oxyanion experiments, we show that strain *Vh*-14B precipitated high Se NP, more than strain 6A (Fig. 3), suggesting that it may be a good candidate for Se NP synthesis. We thought tellurite might accelerate this process. Unfortunately, tellurite addition actually decreased Se precipitation (Fig. SI-4). This can probably be explained by the strain’s low Te resistance.

If microbes are used for industrial scale production of Te-NP and Se-NP, the microbial production of alkylated metalloids is concerning because they have serious health and environmental effects from chronic and acute exposure. For all strains, we show here that mixtures of Se and Te in liquid cultures dramatically inhibited Se and Te volatilization. This result is different from previous studies showing that Se stimulated the formation of methylated Te forms in liquid cultures (44, 66). In the presence of tellurite, the decrease in the production of volatile Se compounds is more pronounced for strain *Vh*-14B (low tellurite resistant) and, to a lesser extent, for strain *Bac*_sp-28A (moderate tellurite resistant) suggesting that the toxicity of tellurite influences Se volatilization. The production of volatile Se compounds by strain *Bac_*sp-6A (moderate tellurite resistant) is less affected by the presence of tellurite, probably due to the protective effect of selenite for this specific strain as discussed above. In our marine yeast and bacteria, the production of volatile Se compounds in the presence of Te appears, however, to be positively correlated with tellurite resistance (Fig. 4b). Furthermore, the presence of mixture in the liquid cultures modifies the nature of volatile Se compounds produced by *Rm*-1A, -13B, -30B, and *Bac*_sp-6A. It favors the production of light volatile Se compounds (DMSe) and inhibits the production of mixed species with sulfur and Se (DMSSe and DMDSSe). The differences between prior studies (44, 66) and the data here may reflect differences in mechanisms of volatilization and/or responses to selenite and tellurite. Much more work is required to establish this for the strains here including genome sequencing, identification of genes that are required for resistance by mutagenesis, and the identification of proteins induced by oxyanion exposure and/or associated with Se and Te NPs. These data would significantly improve our knowledge of the diversity of mechanisms involved in the precipitation and volatilization of Se and Te.

In conclusion, our results show that all strains studied here precipitate and volatilize Se and Te, but the fate of Se and Te will depend on the specific micro-organisms and microbial communities. The differences between this study and those conducted on other micro-organisms further reinforce the idea that there is not yet a consistent model that allows for prediction of Se or Te fate directly from microbial community composition. For the marine strains here, non-aerated cultures of *Rm*-1A, -13B, and -30B strains and strain *Bac_*sp-6A efficiently precipitated Te but not Se, while strains *Bac*_sp-28A and *Vh*-14B precipitated high amounts of Se. Microbial production of Te and Se NPs should account for the production of toxic volatile compounds shown by our work. This could severely limit adoption of microbial NP production. Here, we show that the presence of mixtures of oxyanions modifies the fate of Te and Se in cultures, thereby the production of Se-containing and Te-containing precipitates (presumably as NPs based on prior work with these strains [49]). For strain *Rm*-13B (and other *Rm* strains), mixtures of Se and Te boost the production of Te precipitates and decreases the production of volatile Te and Se compounds. An excess of selenite is not necessary to trigger this mechanism, a low concentration of selenite is sufficient. This trend is more pronounced for strain *Bac_*sp-6A, probably due to the protective effect of selenite increasing Te resistance. Moreover, strain *Bac_*sp-6A precipitates high amount of Se precipitates in the presence of mixtures. While additional work is needed to confirm that the enhanced precipitation is nanoparticulate and verify NP composition, the data here show that combining Se and Te has significant potential to increase efficiency of microbial Se/Te precipitation while significantly limiting the negative outcome of volatilization. Such results open up the possibility to exploit *Rhodotorula mucilaginosa strain* 13B and *Bacillus* sp. strain 6A as a green alternative for the synthesis of Se NPs and Te NPs.

## MATERIALS AND METHODS

### Media and reagents

Chemicals (e.g., Na_2_SeO_3_, Na_2_TeO_3_) and reagents were purchased from SigmaAldrich (St. Louis, MO), Fisher Scientific (Pittsburgh, PA), or VWR Scientific (West Chester, PA) and were of the highest grade available. High-purity HNO_3_ was prepared from trace metal-grade HNO_3_ in a quartz subboiler (Quartz & Silice, Courbevoie, France). The growth medium for all experiments was LB-marine prepared as previously described (49). The medium was solidified with 1.5% (wt/vol) biotechnology-grade agar (Fisher Scientific) for colony isolation on streak plates. All growth on plates was carried out at room temperature, while liquid cultures were grown at 30°C with shaking at 250 rpm.

### Selenite resistance determination

The viability of cells following exposure to selenite was assessed by growing each strain in LB-marine medium in the absence of selenite and by quantifying their ability to grow on LB-marine agar containing selenite. Cell concentrations in liquid cultures were determined by direct counting using a Hausser counting chamber (Fisher Scientific, Pittsburgh, PA). Cultures were diluted to a concentration of 2 × 10^3^ cells mL^−1^ in LB-marine medium. One hundred microliters of this suspension (200 CFU) was spread onto LB-marine agar without amendment (control) or containing concentrations of Na_2_SeO_3_ ranging from 75 to 1,200 µg mL^−1^ (0 to 6.9 mM). Plates were incubated for at least 2 weeks to allow for the observation of slow-growing colonies.

### Experiment set-up

*R. mucilaginosa* (strains 1A, 13B and 30B), *Bacillus* sp. (strains 6A and 28A), and *V. halodenitrificans* (strain 14B) were grown in aerated and non-aerated liquid cultures in the presence of tellurite (0.68 mM), selenite (0.68 mM), or tellurite-selenite mixtures of 0.68 mM(Te):0.068 mM(Se), 0.34 mM(Te):0.34 mM(Se), or 0.068 mM(Te):0.68 mM(Se) (i.e., 10:1, 1:1, or 1:10). For non-aerated liquid cultures, 2 mL of medium was placed in sealed 18 mL headspace vials (Supelco, Bellefonte, PA) with the indicated concentrations of oxyanion and an initial headspace of room air at ambient pressure as described in reference 49. Individual cultures were sacrificed after 4, 8, 14, and 35 days to quantify precipitated, soluble, and volatile species.

For aerated liquid cultures, a “train”-style apparatus for growing cultures with continuous aeration while trapping volatile compounds was used as described by (50). Cultures (10 mL) were immersed in a 30°C water bath and connected to a downstream series of tubes by Teflon tubing passed through silicone rubber stoppers (Fig. SI-8). During culturing, room air filtered through a 0.20 µm membrane filter (Millipore, Billerica, MA) was bubbled into the culture solution to move headspace gas through the system. The volumetric airflow rate was 1.4 ± 0.2 mL min^−1^. The air stream was then passed through a cotton layer (ca. 7 cm in length) in a test tube to remove aerosol droplets and then bubbled through three successive 10 mL volumes of 7.5 N HNO_3_ in 20 mL glass vials to trap volatile compounds. No Te or Se was recovered from any of the traps when sterile medium was used instead of culture in the system. Precipitated, soluble, and volatile species were quantified after 8 days for all strains. For strain *Rm-*13B and strain *Bac_*sp-6A, individual cultures were sacrificed after 4, 8, 14, and 35 days to quantify precipitated, soluble, and volatile species.

### Quantification of solid, dissolved, and volatile tellurium and selenium species

Te and Se content in all samples was determined by graphite furnace atomic absorption spectroscopy as described in references 49 and 50. Briefly, cultures were centrifuged (9,000 × *g*, 25 min). Supernatant was evaporated to dryness at 90–95°C and dissolved with high-purity, sub-boiled HNO_3_, dried at 90–95°C, and dissolved in 5% (vol/vol) HNO_3_. The cell pellet was washed with Te- and Se-free medium and dissolved with sub-boiled HNO_3_, dried as above, and dissolved in 5% (vol/vol) HNO_3_. Trap samples were evaporated to dryness at 90–95°C. The residue was dissolved with sub-boiled HNO_3_, dried at 90–95°C, and dissolved in 5% (vol/vol) HNO_3_. A hollow cathode lamp was employed as the emission source at 214.3 nm with a slit width of 2 nm and 30 mA lamp current for Te. The emission source wavelength was 196 nm with a slit width of 2 nm and 25 mA lamp current for Se. A matrix modifier containing palladium (30 µg mL^−1^, 0.6 µg Pd) and magnesium (200 µg mL^−1^, 4 µg Mg) was injected into the furnace to prevent the formation of TeO (g) during pyrolysis. For Se, 1 µg Pd mixed with 3 µg Mg was used as matrix modifier. High-purity argon was used as the internal gas. Tubes with pyrolytic graphite coating were used throughout the experiments. Measurements were performed in peak area mode on 20 µL aliquots of the sample. The instrument parameters and the temperature-time program are presented in Table SI-1.

No Te and Se precipitates were found in sterile controls (medium amended with tellurite or selenite) and no Te and Se were detected in traps connected to sterile tellurite-amended medium suggesting that abiotic reactions are not involved in precipitation and volatilization of these compounds.

### Quantification and speciation of volatile Se and Te species

For non-aerated liquid cultures, after 8 days of growth, the headspace above cultures was sampled with a gas-tight syringe and analyzed by gas chromatography-inductively coupled plasma mass spectrometry. Briefly, analyses were performed by highly sensitive head-space sampling and gas chromatography (GC) hyphenated to an inductively coupled plasma mass spectrometer (ICPMS) in a similar way to a previous study (67). The GC-ICPMS (Thermofisher) was preceded by headspace sampling with a gas-tight syringe to collect volatile Se and Te species present in the headspace above the incubated culture. Once sampling was completed, the analytes were directly injected in the heated GC injection port for GC-ICPMS analysis. Quantification was performed according to reference 68 using commercially available gaseous Se and Te standards. Identification of the Se and Te compounds associated with unknown chromatographic peaks was then carried out based on GC retention times (tR) in reference to the known boiling points (Bp) of other methylated Group *Bac_*sp-6A congeners such as dimethyl sulfide (CH_3_SCH_3_; DMS), dimethyl selenide (CH_3_SeCH_3_; DMSe), and DMTe as previously done (68). Figure S9 demonstrates the highly linear relation obtained between tR and referenced Bp requisite for confirming volatile organo-chalcogen standards or compounds identified in the cultures and under the same analytical conditions.

## SUPPLEMENTAL MATERIAL

Supplemental material aem.00881-25-s0001.docx Table SI-1; Fig. SI-1 to SI-9.

ASM does not own the copyrights to Supplemental Material that may be linked to, or accessed through, an article. The authors have granted ASM a non-exclusive, world-wide license to publish the Supplemental Material files. Please contact the corresponding author directly for reuse.
