# Supplementary material for: Tellurite and selenite processing by tellurite resistant marine microbes
Source: Appl Environ Microbiol. 2025 Oct 1;91(10):e00881-25. doi: 10.1128/aem.00881-25 (PMC12542773; doi:10.1128/aem.00881-25)
Supplement: Supplemental material — Table SI-1; Fig. SI-1 to SI-9. [file aem.00881-25-s0001.docx]

**Supporting information**

Responses of tellurite resistant microbes to selenite and mixtures

SI-1:

For *Bacillus* sp. strain 28A and *V. halodenitrificans* strain 14B, all volatile Se species decreased in the presence of Te. In contrast to the other strains, DMSe always remained at < 5% of Se volatiles even at high tellurite:selenite ratios. Mixed species were always the dominant volatile Se species produced by these strains, between 61% and 90% for strain *Bac_*sp-28A and between 77% and 99% for strain *Vh-*14B.

Table SI-1: Experimental conditions used for the determination of selenium by GF-AAS

Fig SI-1: Volatilization and precipitation rates of Te and Se (in µM/d) in aerated liquid cultures of strains *Rm-*13B and *Bac_*sp-6A exposed to 0.68mM SeO_3_^2-^ or 0.68mM TeO_3_^2-^ as a function of time. The data presented were calculated using results obtained from individual experiments performed for each strain.

Figure SI-2: Te and Se volatile compounds in headspace of non-aerated liquid cultures amended with (a) Te (0.68 mM) and b) Se (0.68 mM). The data presented were obtained from individual experiments performed for each strain.

Fig SI-3: Soluble (blue bars), particulate (dark bars) and volatile (yellow bars) Se (top) and Te (bottom) in aerated liquid cultures of strains *Rm-*13B and *Bac_*sp-6A exposed to 0.68mM SeO_3_^2-^, 0.68mM TeO_3_^2-^ and mixtures SeO_3_^2-^ and TeO_3_^2-^(0.68mM(Te):0.068mM(Se), 0.34mM(Te):0.34mM(Se) or 0.068mM(Te):0.68mM(Se) ratios, i.e., 10:1, 1:1 or 1:10 ratios). The data presented were obtained from individual experiments performed for each condition and each strain. Bars are the mean of four measurements (± the standard deviation).

Fig SI-4: Soluble (blue bars) and particulate (dark bars) Se (top) and Te (bottom) in strains, after 8 days of growth, in non-aerated liquid cultures exposed to SeO_3_^2-^, TeO_3_^2-^ and mixtures SeO_3_^2-^ and TeO_3_^2-^. 0.68mM SeO_3_^2-^, 0.68mM TeO_3_^2-^ and various mixtures SeO_3_^2-^ and TeO_3_^2-^ (0.68mM(Te):0.068mM(Se), 0.34mM(Te):0.34mM(Se) or 0.068mM(Te):0.68mM(Se) ratios, i.e., 10:1, 1:1 or 1:10 ratios) were added to cultures of strain 28A, while 0.17mM SeO_3_^2-^, 0.17mM TeO_3_^2-^ and various mixtures SeO_3_^2-^ and TeO_3_^2-^ (0.17mM(Te):0.017mM(Se), 0.085mM(Te):0.085mM(Se) or 0.017mM(Te):0.17mM(Se) ratios) were added to cultures of strain *Vh-*14B. The data presented were obtained from individual experiments performed for each condition and each strain. Bars are the mean of four measurements (± the standard deviation).

Fig SI-5: Volatile Se, Te and mixed (with S) species in headspace of non-aerated liquid cultures amended with 0.68mM SeO_3_^2-^, 0.68mM TeO_3_^2-^ and various mixtures SeO_3_^2-^ and TeO_3_^2-^ (0.68mM(Te):0.068mM(Se), 0.34mM(Te):0.34mM(Se) or 0.068mM(Te):0.68mM(Se) ratios, i.e., 10:1, 1:1 or 1:10 ratios) (log scale). The data presented were obtained from individual experiments performed for each condition and each strain.

Fig SI-6: Relative abundance (i.e., the abundance of each species relative to the total concentration of volatile Se/Te, in %) of Se and mixed Se/S species in headspace of cultures amended with 0.68mM SeO_3_^2-^, 0.68mM TeO_3_^2-^ and various mixtures SeO_3_^2-^ and TeO_3_^2-^ (0.68mM(Te):0.068mM(Se), 0.34mM(Te):0.34mM(Se) or 0.068mM(Te):0.68mM(Se) ratios, i.e., 10:1, 1:1 or 1:10 ratios). The data presented were calculated using the results obtained from individual experiments performed for each condition and each strain.

Fig SI-7: Relative abundance (i.e., the abundance of each species relative to the total concentration of volatile Se/Te, in %) of Te and mixed Te/S species in headspace of cultures amended with 0.68mM SeO_3_^2-^, 0.68mM TeO_3_^2-^ and various mixtures SeO_3_^2-^ and TeO_3_^2-^ (0.68mM(Te):0.068mM(Se), 0.34mM(Te):0.34mM(Se) or 0.068mM(Te):0.68mM(Se) ratios, i.e., 10:1, 1:1 or 1:10 ratios). The data presented were calculated using the results obtained from individual experiments performed for each condition and each strain.

Fig SI-8: Culture apparatus for growth of strains with continuous aeration while trapping volatile Te compounds. Cultures were maintained at 30°C in a water bath, and all other components were at room temperature (adapted from (50)).

Fig SI-9: Correlations between literature and calculated boiling points and retention time by gas chromatography (GC) for volatile sulphur, selenium and tellurium species (standards, references and identified compounds).

| Step | Temperature (°C) | Ramps (time/s) | Hold (time/s) | Argon flow (ml/min) |
| --- | --- | --- | --- | --- |
| Drying | 100 | 5 | 20 | 250 |
| Drying | 140 | 15 | 15 | 250 |
| Pyrolysis | 600 | 10 | 20 | 250 |
| Atomization | 2100 | 0 | 5 | 0 |
| Clean out | 2600 | 1 | 3 | 250 |
|  |  |  |  |  |
| Instrumental parameters | |  |  |  |
| Wavelength (nm) | 196 |  |  |  |
| Slit width (nm) | 2 |  |  |  |
| Source | Se hollow cathode lamp | |  |  |
| Read time (s) | 5 |  |  |  |
| Signal mode | Peak area |  |  |  |

Table SI-1: Experimental conditions used for the determination of selenium by GF-AAS


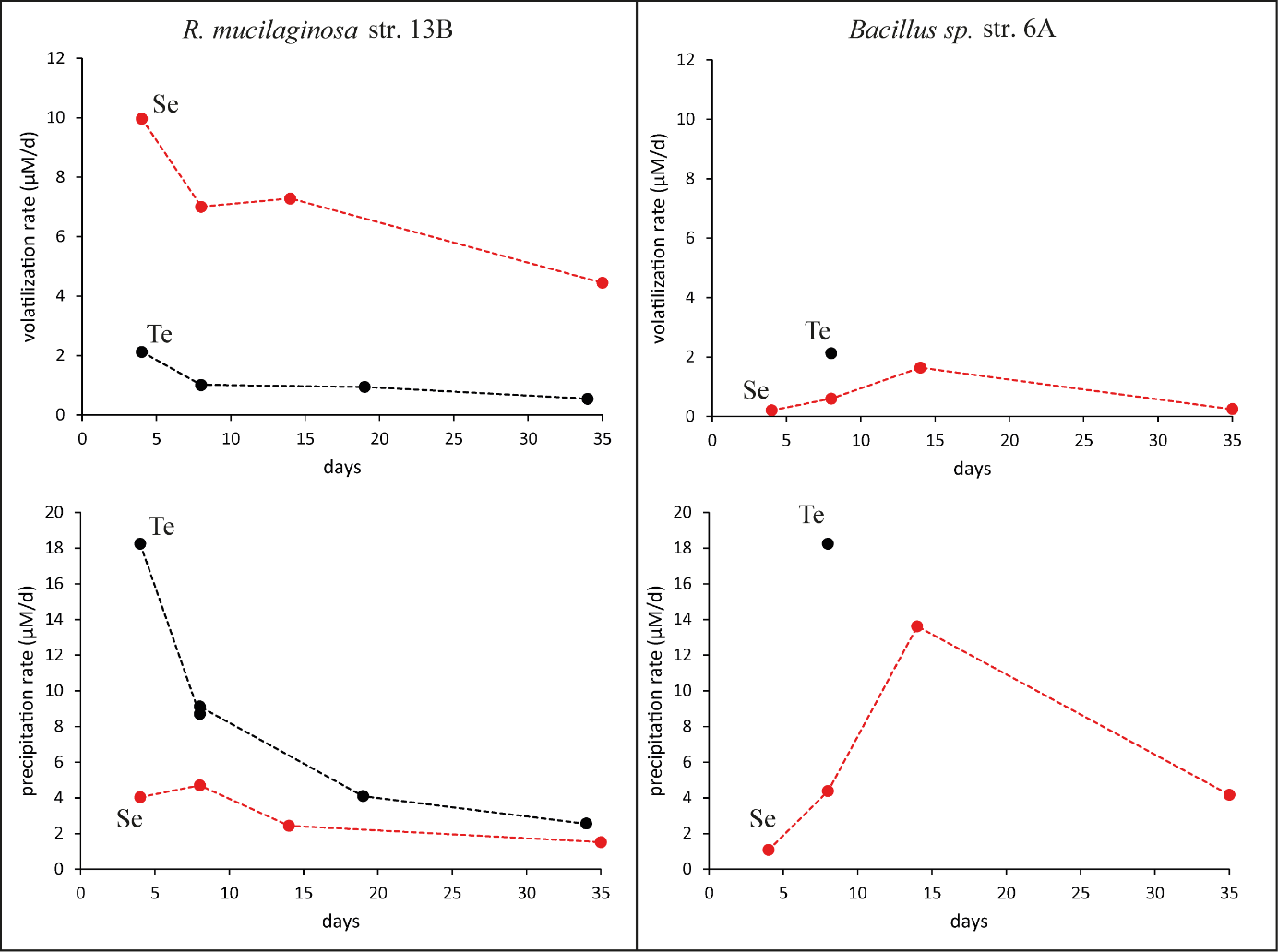


Fig SI-1: Volatilization and precipitation rates of Te and Se (in µM/d) in aerated liquid cultures of strains *Rm-*13B and *Bac_*sp-6A exposed to 0.68mM SeO_3_^2-^ or 0.68mM TeO_3_^2-^ as a function of time. The data presented were calculated using results obtained from individual experiments performed for each strain.


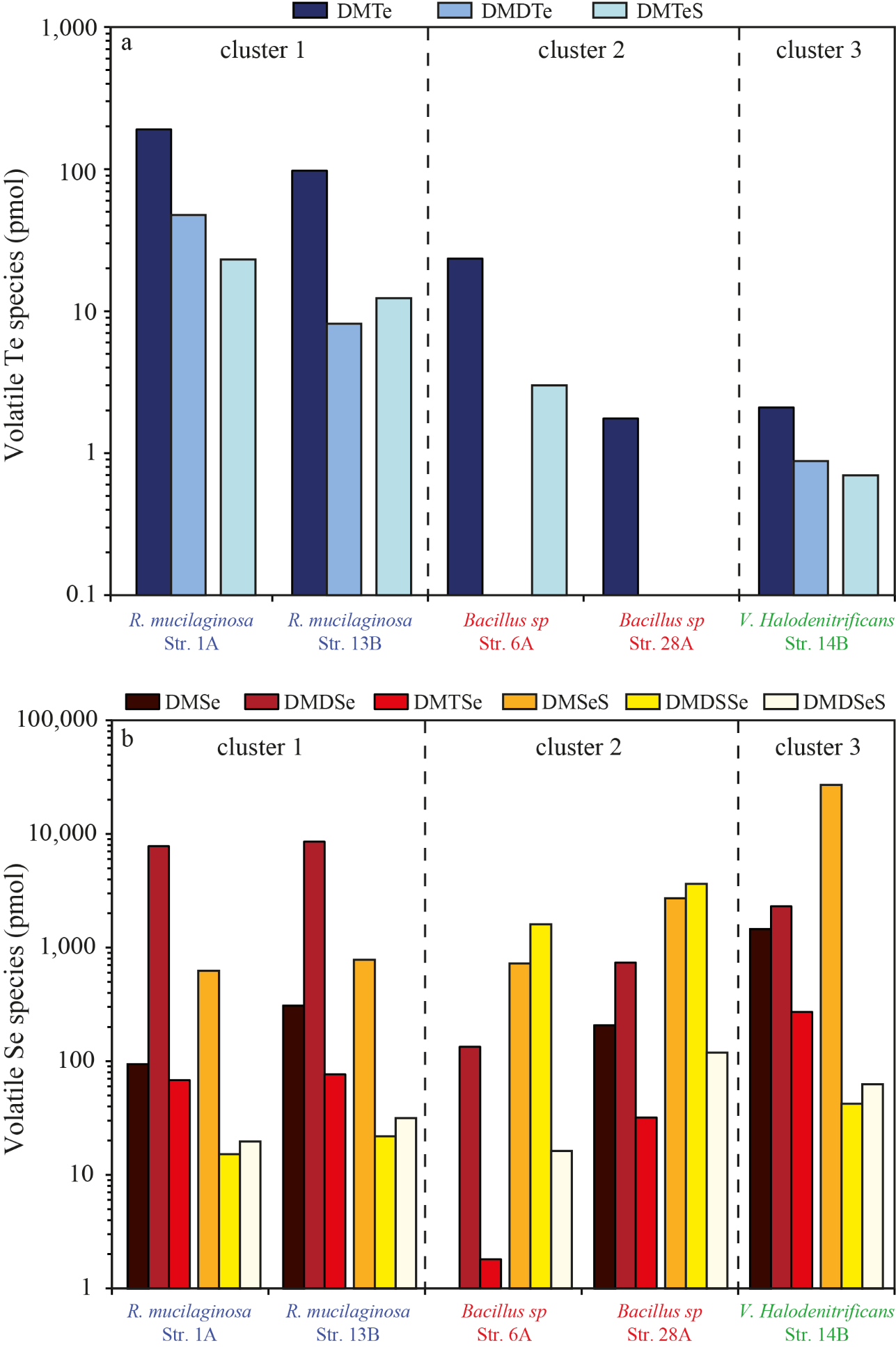


Figure SI-2: Te and Se volatile compounds in headspace of non-aerated liquid cultures amended with (a) Te (0.68 mM) and b) Se (0.68 mM). The data presented were obtained from individual experiments performed for each strain.


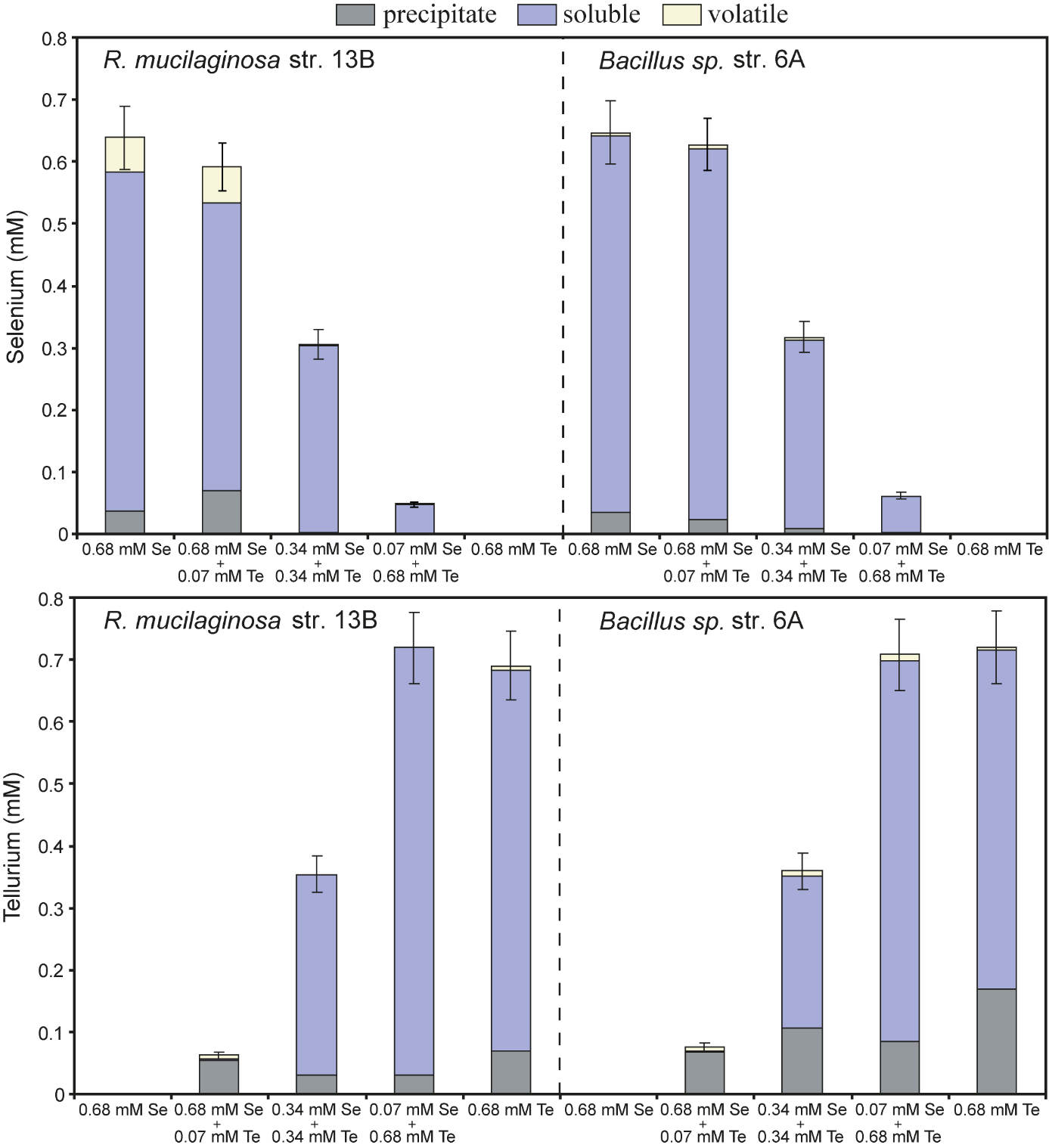


Fig SI-3: Soluble (blue bars), particulate (dark bars) and volatile (yellow bars) Se (top) and Te (bottom) in aerated liquid cultures of strains *Rm-*13B and *Bac_*sp-6A exposed to 0.68mM SeO_3_^2-^, 0.68mM TeO_3_^2-^ and mixtures SeO_3_^2-^ and TeO_3_^2-^(0.68mM(Te):0.068mM(Se), 0.34mM(Te):0.34mM(Se) or 0.068mM(Te):0.68mM(Se) ratios, i.e., 10:1, 1:1 or 1:10 ratios). The data presented were obtained from individual experiments performed for each condition and each strain. Bars are the mean of four measurements (± the standard deviation).


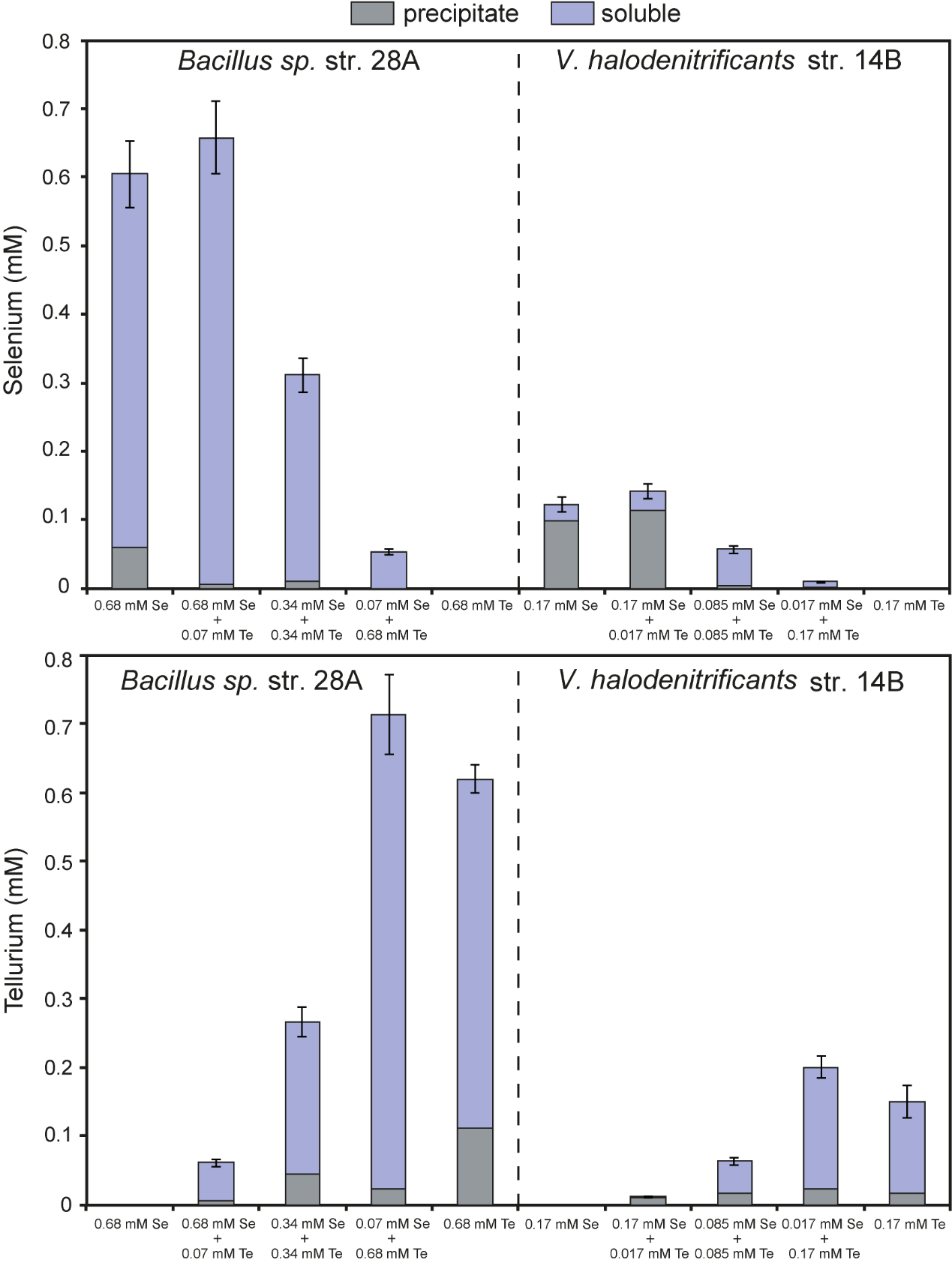


Fig SI-4: Soluble (blue bars) and particulate (dark bars) Se (top) and Te (bottom) in strains, after 8 days of growth, in non-aerated liquid cultures exposed to SeO_3_^2-^, TeO_3_^2-^ and mixtures SeO_3_^2-^ and TeO_3_^2-^. 0.68mM SeO_3_^2-^, 0.68mM TeO_3_^2-^ and various mixtures SeO_3_^2-^ and TeO_3_^2-^ (0.68mM(Te):0.068mM(Se), 0.34mM(Te):0.34mM(Se) or 0.068mM(Te):0.68mM(Se) ratios, i.e., 10:1, 1:1 or 1:10 ratios) were added to cultures of strain 28A, while 0.17mM SeO_3_^2-^, 0.17mM TeO_3_^2-^ and various mixtures SeO_3_^2-^ and TeO_3_^2-^ (0.17mM(Te):0.017mM(Se), 0.085mM(Te):0.085mM(Se) or 0.017mM(Te):0.17mM(Se) ratios) were added to cultures of strain *Vh-*14B. The data presented were obtained from individual experiments performed for each condition and each strain. Bars are the mean of four measurements (± the standard deviation).


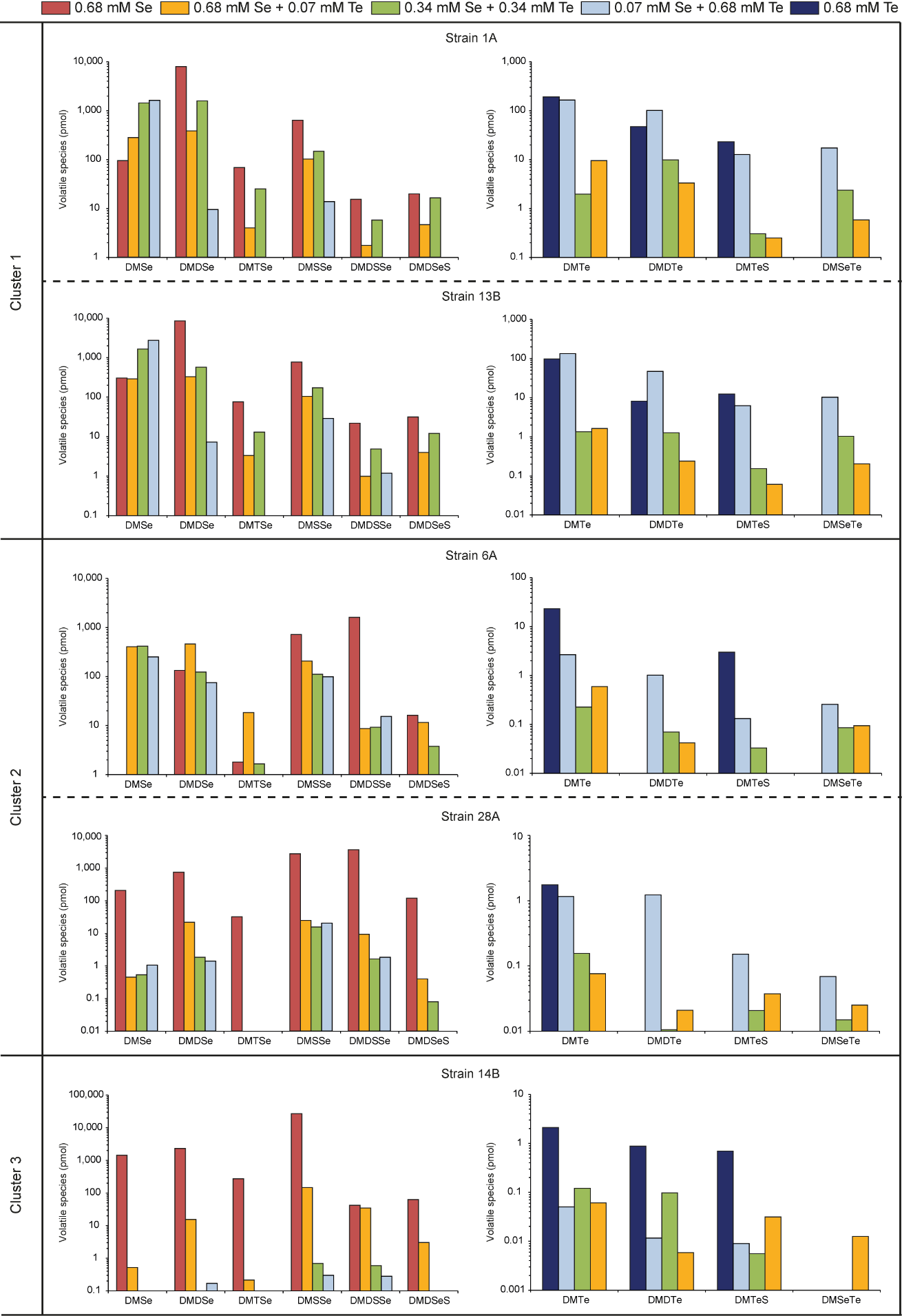


Fig SI-5: Volatile Se, Te and mixed (with S) species in headspace of non-aerated liquid cultures amended with 0.68mM SeO_3_^2-^, 0.68mM TeO_3_^2-^ and various mixtures SeO_3_^2-^ and TeO_3_^2-^ (0.68mM(Te):0.068mM(Se), 0.34mM(Te):0.34mM(Se) or 0.068mM(Te):0.68mM(Se) ratios, i.e., 10:1, 1:1 or 1:10 ratios) (log scale). The data presented were obtained from individual experiments performed for each condition and each strain.


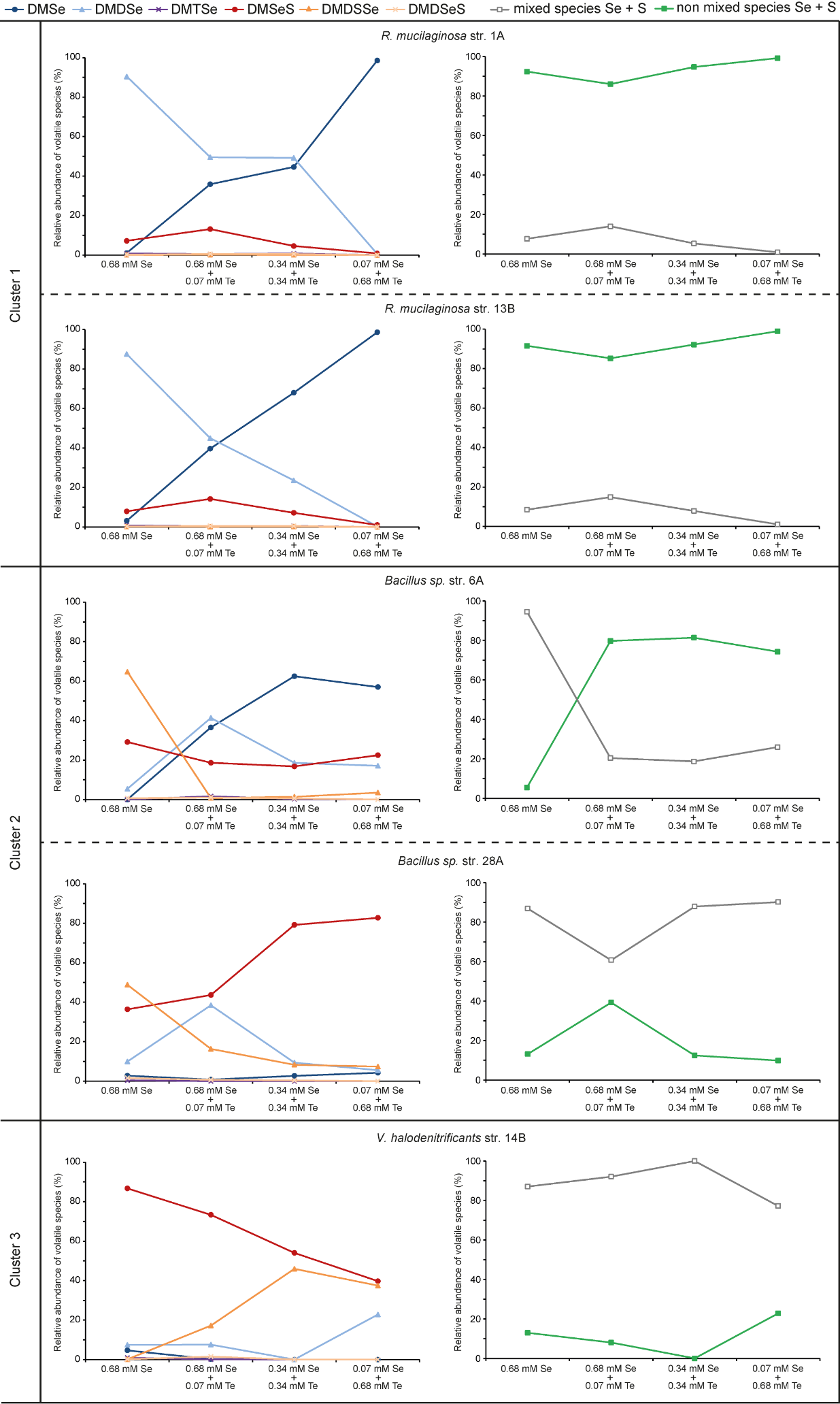


Fig SI-6: Relative abundance (i.e., the abundance of each species relative to the total concentration of volatile Se/Te, in %) of Se and mixed Se/S species in headspace of cultures amended with 0.68mM SeO_3_^2-^, 0.68mM TeO_3_^2-^ and various mixtures SeO_3_^2-^ and TeO_3_^2-^ (0.68mM(Te):0.068mM(Se), 0.34mM(Te):0.34mM(Se) or 0.068mM(Te):0.68mM(Se) ratios, i.e., 10:1, 1:1 or 1:10 ratios). The data presented were calculated using the results obtained from individual experiments performed for each condition and each strain.


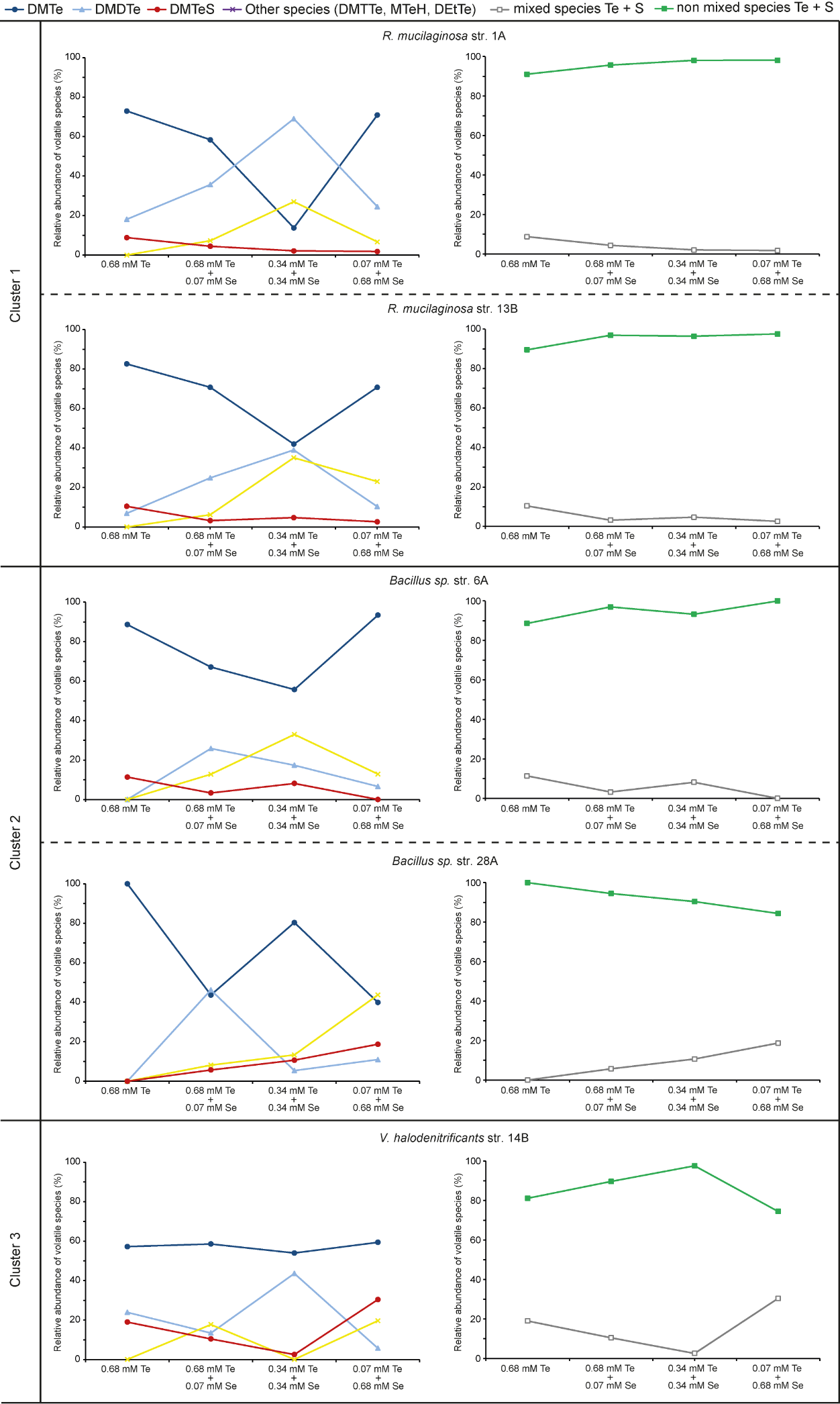


Fig SI-7: Relative abundance (i.e., the abundance of each species relative to the total concentration of volatile Se/Te, in %) of Te and mixed Te/S species in headspace of cultures amended with 0.68mM SeO_3_^2-^, 0.68mM TeO_3_^2-^ and various mixtures SeO_3_^2-^ and TeO_3_^2-^ (0.68mM(Te):0.068mM(Se), 0.34mM(Te):0.34mM(Se) or 0.068mM(Te):0.68mM(Se) ratios, i.e., 10:1, 1:1 or 1:10 ratios). The data presented were calculated using the results obtained from individual experiments performed for each condition and each strain.


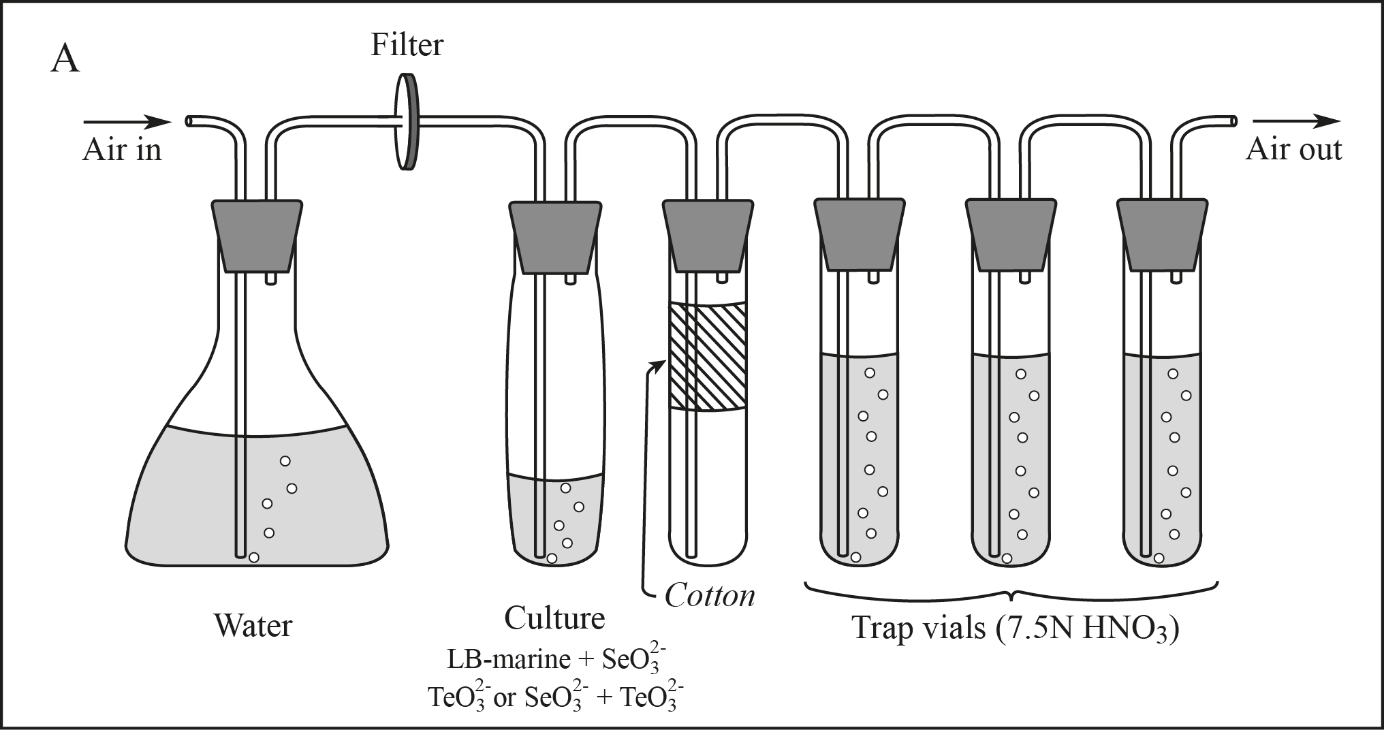


Fig SI-8: Culture apparatus for growth of strains with continuous aeration while trapping volatile Te compounds. Cultures were maintained at 30°C in a water bath, and all other components were at room temperature (adapted from (50)).


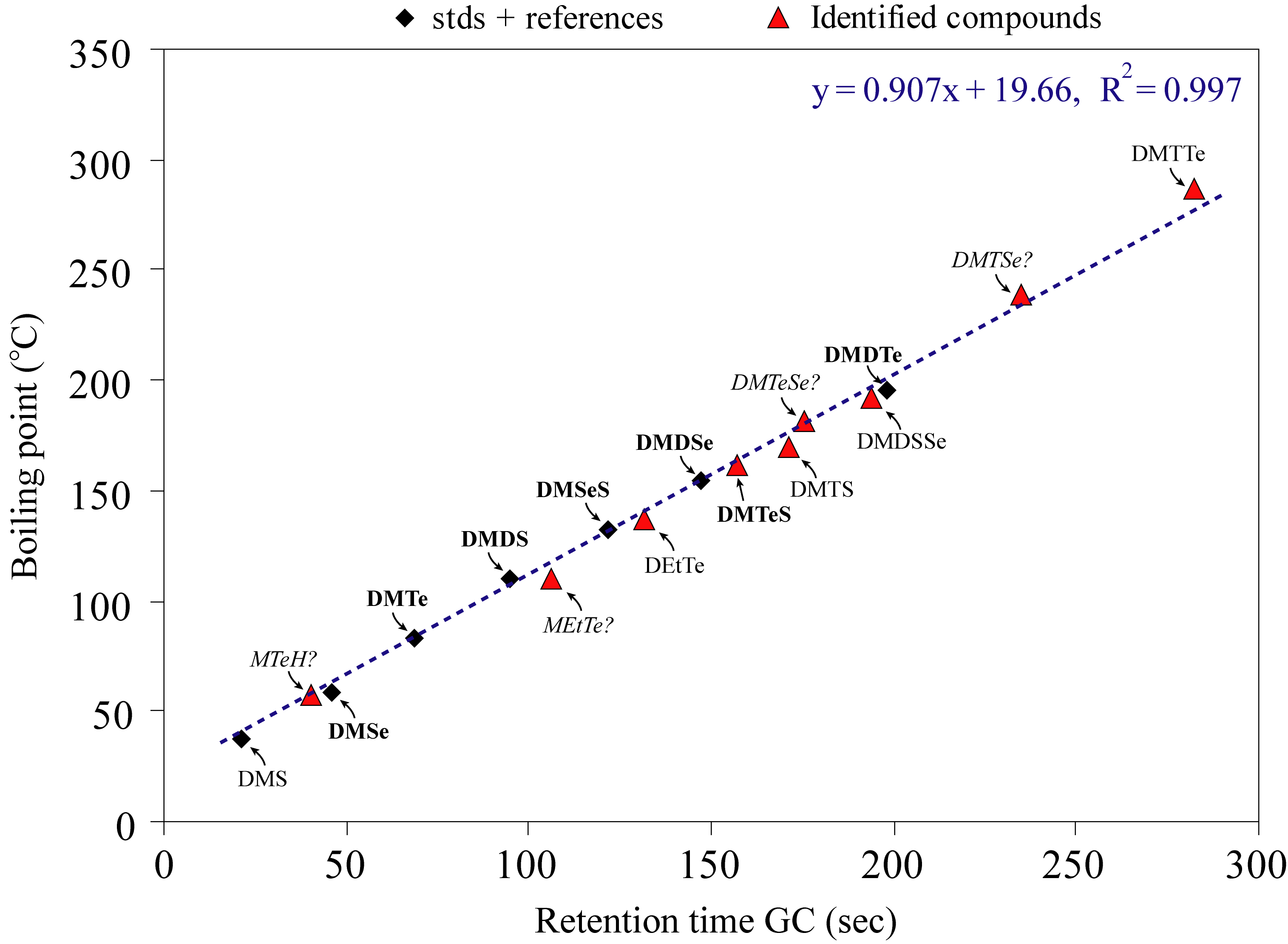


Fig S9: Correlations between literature and calculated boiling points and retention time by gas chromatography (GC) for volatile sulphur, selenium and tellurium species (standards, references and identified compounds).
